# Bridging oral and systemic health: exploring pathogenesis, biomarkers, and diagnostic innovations in periodontal disease

**DOI:** 10.1007/s15010-025-02568-y

**Published:** 2025-05-26

**Authors:** Max Foroughi, Mahmoud Torabinejad, Nikola Angelov, David M. Ojcius, Keykavous Parang, Marcus Ravnan, Jerika Lam

**Affiliations:** 1 Department of Preventive and Restorative Dentistry, Arthur A. Dugoni School of Dentistry, University of the Pacific, 155 Fifth Street, San Francisco, CA 94103 USA; 2 Department of Endodontics, School of Dentistry, Loma Linda University School of Dentistry, Loma Linda, CA USA; 3 Department of Periodontics and Dental Hygiene, The University of Texas Health Science Center at Houston School of Dentistry, Houston, TX USA; 4 Department of Biomedical Sciences, Arthur A. Dugoni School of Dentistry, University of the Pacific, San Francisco, CA USA; 5 Department of Biomedical and Pharmaceutical Sciences, Center for Targeted Drug Delivery, Chapman University School of Pharmacy, Harry and Diane Rinker Health Science Campus, Irvine, CA USA; 6 Thomas J. Long School of Pharmacy and Health Sciences, University of the Pacific, Stockton, CA USA; 7 Department of Pharmacy Practice, School of Pharmacy, Chapman University, Irvine, CA USA

**Keywords:** Periodontal diseases, Systemic health, Biomarkers, Diagnostic, Lateral flow assays, Oral infection

## Abstract

**Purpose:**

This narrative review explores the multifaceted links between periodontal diseases (gingivitis and periodontitis) and systemic health conditions, including cardiovascular disease, diabetes, adverse pregnancy outcomes, Alzheimer’s disease, cancers, rheumatoid arthritis, and respiratory infections. It aims to synthesize evidence on how local oral infections exert systemic effects and evaluate the potential of diagnostic technologies to monitor these interactions.

**Methods:**

This narrative review synthesizes current scientific literature on periodontal disease pathogenesis, focusing on key pathogens (e.g., *Porphyromonas gingivalis*, *Fusobacterium nucleatum*) and their roles in driving local and systemic inflammation via virulence factors and microbial dysbiosis. It examines biomarker-based diagnostic approaches (e.g., IL-1β, TNF-α, microbial DNA) in saliva, blood, and gingival crevicular fluid (GCF) and evaluates current and emerging diagnostic tools (e.g., ELISA, PCR, lateral flow assays, biosensors, microfluidics).

**Results:**

The review highlights that periodontal pathogens contribute to systemic disease through complex mechanisms including persistent inflammation (driven by cytokines like IL-1β, TNF-α), endotoxemia (via LPS, noting pathogen-specific structural variations impacting immune response), molecular mimicry, and immune modulation. Current diagnostic methods provide valuable information but often face limitations in speed, portability, and multiplexing capability needed for comprehensive point-of-care assessment. Emerging technologies, particularly multiplex platforms integrating biosensors or microfluidics, demonstrate significant potential for rapid, user-friendly analysis of multiple biomarkers, facilitating earlier detection and personalized risk stratification, especially in high-risk populations.

**Conclusion:**

Periodontal diseases significantly impact systemic health via intricate microbial and inflammatory pathways. The complexity of these interactions necessitates moving beyond conventional diagnostics towards integrated, advanced technologies. Implementing rapid, multiplex biomarker detection platforms within a multidisciplinary healthcare framework holds the potential to revolutionize early detection of linked conditions, improve personalized management strategies, and ultimately reduce the systemic burden of periodontal disease.

## Introduction

Periodontal diseases, including gingivitis and periodontitis, are highly prevalent chronic inflammatory conditions globally [1]. Severe periodontitis, the advanced form of the disease, was ranked as the sixth most prevalent disease globally based on earlier estimates [2], and updated data for 2019 indicate approximately 1.1 billion prevalent cases worldwide (95% uncertainty interval: 0.8–1.4 billion). Between 1990 and 2019, the age‐standardized prevalence rate of severe periodontitis increased by 8.44% (range 6.62–10.59%), with a disproportionately higher burden in less developed regions, significantly driven by global population growth [3]. In the United States, national survey data from 2009 to 2012 indicated an alarmingly high prevalence, with approximately 47.2% of adults aged 30 years and older affected by some form of periodontitis, increasing to 70% among those aged 65 and older [4, 5]. These diseases involve inflammation of the gingiva, periodontal ligament, and alveolar bone, often leading to progressive tissue destruction and eventual tooth loss [6, 7].

The impact of periodontal disease extends beyond oral health with established links to various systemic conditions. Risk factors such as smoking, diabetes mellitus, obesity, and low income exacerbate periodontal disease severity and contribute to the establishment of a dysbiotic oral microbiome, which perpetuates inflammatory processes associated with systemic diseases [8]. The oral cavity serves as a gateway to the body, where an imbalance in microbial flora can trigger systemic inflammation. Pathogens like *Porphyromonas gingivalis*, *Treponema denticola*, and *Fusobacterium nucleatum* have been detected in systemic sites such as atherosclerotic plaques, amniotic fluid, and colorectal tumors, linking them to cardiovascular diseases, adverse pregnancy outcomes, and certain cancers [9–17].

Periodontal diseases contribute to systemic health issues by triggering chronic inflammation, as evidenced by elevated levels of biomarkers like C-reactive protein (CRP), interleukin-6 (IL-6), and tumor necrosis factor-alpha (TNF-α) [18]. This inflammation can worsen conditions such as diabetes mellitus and coronary heart disease [19, 20]. Furthermore, the economic impact of periodontal disease underscores its significance as a public health concern. A study published in the Journal of Periodontology estimated that, in 2018, the United States incurred approximately $3.49 billion in direct costs for the diagnosis and treatment of periodontal disease. Additionally, indirect costs, primarily due to lost productivity, were estimated at $150.57 billion. Collectively, these figures underscore the substantial economic burden posed by periodontitis [21]. For example, oral health issues, including periodontal diseases, are responsible for the loss of millions of work hours annually, affecting both individuals and the healthcare system. Roughly $46 billion dollars of U.S. productivity (2015 U.S. dollars) is lost yearly due to untreated oral diseases [22]. Despite advancements in diagnostic techniques, conventional approaches often involve laboratory tests that are expensive, time-consuming, and rely on highly technical equipment. To address these challenges, innovative diagnostic tools such as lateral flow assays, microfluidic devices, and biosensors have emerged as promising alternatives [23, 24]. The assessment of specific biomarkers in oral fluids offers a non-invasive and effective approach for the diagnosis and monitoring of periodontal diseases. Artificial Intelligence (AI) is increasingly being utilized in periodontology to enhance diagnostic accuracy and efficiency. AI, particularly through the use of convolutional neural networks (CNNs), has been applied to analyze visual imaging data for the diagnosis of periodontal diseases. This review emphasizes the potential of AI to assist clinicians in diagnosing and treating periodontitis by improving the interpretation of complex data [25]. These developments underscore the transformative role of AI in periodontal diagnostics, particularly in the assessment of biomarkers and imaging data, leading to more accurate and efficient disease detection and management. AI is significantly enhancing periodontal diagnostics by improving the speed and accuracy of biomarker assessment. AI platforms can simultaneously analyze multiple biomarkers, identify disease patterns, and provide real-time diagnostic recommendations, thereby enhancing efficiency and accessibility. A narrative review published in the Periodontology 2000 journal discusses the role of AI in personalized diagnostics within periodontology, highlighting its potential to analyze diverse data sets, including clinical records, imaging, and molecular information, to tailor diagnostic strategies and improve precision in periodontal care [26].

This review explores the link between periodontal and systemic health, emphasizing the potential of biomarkers and advanced diagnostic technologies. By addressing challenges in diagnosis and proposing strategies for early detection, this review highlights the critical need for an integrated approach for managing periodontal and systemic diseases.

### Literature search approach

This manuscript is presented as a narrative review. To gather the information synthesized herein, a general literature search was conducted using primary databases including PubMed/Medline, Scopus, and Google Scholar. Key concepts and search terms related to periodontal disease, specific systemic conditions (e.g., cardiovascular disease, diabetes mellitus, Alzheimer's disease, etc.), relevant pathogens (e.g., Porphyromonas gingivalis, Fusobacterium nucleatum), biomarkers (e.g., IL-6, CRP, MMPs), and diagnostic technologies (e.g., biosensors, microfluidics, lateral flow assays) were employed. Consistent with the goals of a narrative synthesis aiming for a broad overview, the selection of references focused on landmark studies, recent advancements, relevant reviews, and representative evidence pertinent to the discussion.

## Pathogenesis of periodontal diseases

Periodontal diseases are chronic inflammatory conditions influenced by the host immune response and alterations in the oral microflora [27–29]. These diseases are primarily initiated by biofilms containing pathogenic bacteria that evade the host's defense mechanisms, leading to inflammation and tissue destruction [30]. The pathogenesis of periodontal disease encompasses both local soft tissue pathology and systemic effects resulting from the dissemination of microorganisms and modulation of the host immune response [27, 31, 32].

### Role of biofilms and key pathogens

Dental biofilms are structured communities of microorganisms embedded in an extracellular matrix that adheres to tooth surfaces [33]. This biofilm structure provides bacteria with protection against host immune responses and antimicrobial agents, enabling their persistence and contribution to disease processes [34, 35]. Microbial dysbiosis, a central element in periodontal disease, refers to an imbalance or disruption in the composition and function of the microbiome. Crucially, it is characterized by a shift not merely in the types of bacteria present but fundamentally in their relative abundance, overall community structure, and collective functional output (e.g., altered metabolic activity and increased virulence factor expression). This shift moves the subgingival environment from a health-associated state, typically dominated by commensal Gram-positive organisms, towards a disease-associated state characterized by a predominance of Gram-negative anaerobes. These pathogens release virulence factors, including toxins, that trigger and intensify inflammatory responses [36].

While microbial dysbiosis is central to periodontal disease, understanding the specific changes within the oral microbiome is critical. In a healthy oral cavity, commensal species, such as *Streptococcus* and *Actinomyces*, dominate and maintain homeostasis by modulating the immune response and outcompeting harmful pathogens [10]. In contrast, periodontal disease is marked by the proliferation of pathogenic species like *Porphyromonas gingivalis*, *Treponema denticola*, and *Fusobacterium nucleatum*, which thrive under these dysbiotic conditions characterized by altered community dynamics and function [9].

Microbiome variability among individuals significantly influences susceptibility to and progression of periodontal disease. Factors such as genetics, diet, smoking, and systemic health play a vital role in shaping the microbiome's composition and function, resulting in unique microbial profiles [37]. For instance, smokers tend to harbor higher proportions of *Treponema* and *Prevotella* species, correlating with increased disease severity [38]. Furthermore, the concept of a "healthy microbiome" is complex, as variations can be observed even among periodontally healthy individuals [29, 39]. Future research aimed at defining microbial signatures of health and disease, considering both composition and functional shifts, is critical for identifying reliable biomarkers and developing personalized diagnostic and therapeutic approaches.

#### Porphyromonas gingivalis

- *P. gingivalis* is a gram-negative, anaerobic bacterium which disturbs the balance of the biofilm community and contributes to bacterial dysbiosis, thus leading to the progression of periodontal disease. Some of its virulence factors include gingipains (cysteine proteases), which digest host proteins, compromise the immune system, and promote tissue invasion [9].
- It expresses a weakly immunogenic lipopolysaccharide (LPS), modifies Toll-like receptor (TLR) signaling and hampers neutrophil killing [40].

#### Treponema denticola

- *T. denticola* is a Gram-negative, obligate anaerobic motile spirochete that is associated with late-stage periodontitis. It forms biofilms that protect the bacteria from the host immune response and antimicrobial treatments. Its dentilisin protease degrades the host extracellular matrix proteins and complements the proteolytic activity of *P. gingivalis* [41].
- This pathogen disrupts epithelial barriers and enables other microorganisms to infect connective tissues [42].

#### Fusobacterium nucleatum

- *F. nucleatum* is another Gram-negative, anaerobic bacterium that acts as a bridge organism in biofilm contributing to the colonization of other pathogens. It adheres to epithelial cells and promotes inflammation by inducing secretion of interleukin-8 (IL-8) and other proinflammatory cytokines [14].
- It can invade tissues and bloodstream and has been linked to diseases such as colorectal cancer and adverse pregnancy outcomes [17].

The virulence mechanisms of key pathogens reveal critical insights into periodontal disease progression. *Porphyromonas gingivalis* employs gingipains to degrade host proteins and modulate the complement system, evading immune detection while promoting inflammation and deeper tissue invasion [9]. Similarly, *Treponema denticola* utilizes dentilisin protease to break down extracellular matrix components and disrupt cell–cell junctions, potentiating the pathogenicity of the biofilm [41]. *Fusobacterium nucleatum* excels in adhesion, with outer membrane adhesins enabling its integration into multispecies biofilms and facilitating colonization of diverse niches [17]. These properties underscore its implication in both local and systemic diseases. Improving our understanding of the oral microbiome's complexity and the specific roles of pathogenic bacteria will help to identify targeted therapies and to develop strategies to restore microbial balance. Future research on microbial interactions and host–pathogen dynamics will further refine periodontal diagnostics and treatment approaches.

### Mechanisms linking oral and systemic health

Periodontal pathogens, such as *Aggregatibacter actinomycetemcomitans* (Aa), *Porphyromonas gingivalis* (Pg), *Tannerella forsythia* (Tf), *Treponema denticola* (Td), *Eubacterium nodatum* (En), *Fusobacterium nucleatum* (Fn), *Prevotella intermedia* (Pi), *Campylobacter rectus* (Cr), *Peptostreptococcus (Micromonas) micros* (Pm), *Eikenella corrodens* (Ec), *Prevotella nigrescens* (Pn), and *Capnocytophaga* species (*gingavalis, ochracea, sputigena*) (Cs), can enter the bloodstream through bacteremia caused by routine activities like chewing, brushing, flossing, and invasive dental procedures [43]. While such bacteremia is typically transient and low-level in individuals with healthy periodontal tissues, efficiently cleared by the host immune system, the situation can be significantly different in periodontitis. In patients with periodontal disease, the inflamed and ulcerated gingival epithelium provides a more persistent portal of entry, and the higher subgingival bacterial load can lead to bacteremia that is more frequent, involves a greater number of microorganisms, and may persist for longer periods [15]. Once in circulation, these pathogens disseminate to tissues and organs, potentially contributing to the pathogenesis of systemic diseases. For instance, the DNA of *Pg* and *Fn* has been identified in atherosclerotic plaques, supporting the hypothesis that oral pathogens are linked to cardiovascular diseases. These findings underscore the ability of periodontal bacteria to spread from the oral cavity to other parts of the body, emphasizing the importance of oral hygiene in preventing systemic diseases [44]. These findings underscore the ability of periodontal bacteria to spread from the oral cavity, particularly in the context of disease, emphasizing the importance of oral hygiene and periodontal health in preventing systemic complications.

Periodontal inflammation contributes to systemic diseases through the release of pro-inflammatory cytokines, such as IL-1β, IL-6, and TNF-α, as well as acute-phase proteins like CRP. These inflammatory mediators exacerbate systemic conditions, including cardiovascular diseases, insulin resistance in diabetes, and adverse pregnancy outcomes [12, 20, 45]. LPS, a major outer membrane component of Gram-negative bacteria, is a key driver of this inflammation. However, the immunostimulatory potential of LPS varies significantly depending on its structure, particularly the lipid A moiety, which interacts with the host Toll-like receptor 4 (TLR4) complex. While classical LPS from bacteria like E. coli possesses a highly pro-inflammatory hexa-acylated lipid A structure, periodontal pathogens such as P. gingivalis produce heterogeneous lipid A forms (often tetra- and penta-acylated) [40, 46]. This structural difference renders P. gingivalis LPS a weak agonist or even an antagonist of human TLR4 [40]. This unique characteristic allows P. gingivalis to potentially subvert or modulate the initial strong inflammatory response typically triggered by LPS, contributing to immune evasion and the establishment of chronic, persistent inflammation rather than acute clearance [46, 47]. LPS released by Gram-negative periodontal pathogens, such as *Pg*, induce endothelial dysfunction, a key event in atherogenesis. LPS activates endothelial cells to express adhesion molecules like E-selectin, intercellular adhesion molecule-1 (ICAM-1), and vascular cell adhesion molecule-1 (VCAM-1), facilitating leukocyte adhesion and migration, which promotes vascular inflammation and plaque formation [27, 48]. Additionally, LPS can disrupt the placental barrier, increasing the risk of preterm births and adverse pregnancy outcomes [16, 49]. Pro-inflammatory cytokines released during periodontal inflammation also play a significant role in systemic metabolic dysregulation. TNF-α inhibits insulin receptor signaling by increasing serine phosphorylation of insulin receptor substrates, impairing glucose uptake in peripheral tissues. Similarly, IL-6 contributes to insulin resistance by enhancing hepatic glucose production and reducing adiponectin levels, an anti-inflammatory cytokine critical for maintaining insulin sensitivity [50, 51]. These interconnected pathways link periodontal inflammation to systemic diseases like diabetes and cardiovascular conditions.

Systemic conditions can also predispose individuals to periodontal disease through mechanisms such as altered immune responses and metabolic dysregulation. For example, diabetes mellitus, particularly when poorly controlled, leads to hyperglycemia-induced impairment of neutrophil chemotaxis and phagocytosis, reducing the host’s ability to combat infection by periodontal pathogens [20, 52]. Elevated glucose levels in gingival crevicular fluid (GCF) create an environment conducive to the growth of pathogenic bacteria, exacerbating periodontal inflammation [53]. Similarly, rheumatoid arthritis, an autoimmune condition, promotes systemic inflammation that weakens the gingival connective tissues and facilitates microbial colonization [54]. Cardiovascular diseases, characterized by chronic low-grade inflammation, may contribute to an inflammatory milieu that accelerates periodontal tissue destruction [55]. Additionally, medications for systemic conditions, such as calcium channel blockers or immunosuppressants, increase susceptibility to periodontal disease by causing gingival overgrowth or dampening immune responses [56].

The host immune response further drives the systemic impact of periodontal disease. Innate immune cells, such as neutrophils, macrophages, and dendritic cells, recognize periodontal pathogens via pattern recognition receptors (PRRs), including Toll-like receptors (TLRs). This triggers the production of pro-inflammatory cytokines, leading to the recruitment of adaptive immune cells like T-helper cells (Th_1_, Th_2_, Th_17_) and B lymphocyte cells. These cells contribute to chronic inflammation and tissue destruction by releasing receptor activator of nuclear factor-kappa B ligand, a protein that promotes osteoclast-mediated bone resorption [10, 47]. Genetic predispositions, such as polymorphisms in cytokine-encoding genes (e.g., IL-1β, TNF-α), heighten inflammatory responses, increasing susceptibility to periodontal and systemic diseases [57]. Molecular mimicry represents another mechanism linking periodontal and systemic health. This occurs when microbial antigens share structural similarities with host proteins, potentially triggering autoimmune responses where the immune system mistakenly attacks host tissues. For example, bacterial Heat Shock Proteins (HSPs), such as HSP60 (GroEL) produced by pathogens like P. gingivalis under stress conditions, exhibit sequence homology with human HSP60. An immune response initially mounted against these bacterial HSPs may subsequently cross-react with human HSPs expressed on host cells, such as endothelial cells in blood vessels (implicated in atherosclerosis) or synovial cells in joints (implicated in rheumatoid arthritis), thereby contributing to inflammation and tissue damage in these systemic conditions [56, 57]. Additionally, virulence factors like gingipains produced by Pg can degrade complement factors, impairing immune clearance mechanisms and perpetuating chronic inflammation, which further amplifies systemic disease progression [46, 58]. Although a growing body of evidence supports associations between periodontal disease and systemic conditions, establishing causation remains challenging [59]. Observational studies demonstrate significant correlations between periodontitis and conditions like cardiovascular disease, diabetes, and adverse pregnancy outcomes; however, confounding factors, such as smoking, socioeconomic status, and comorbidities, complicate these relationships [8, 60]. Reverse causation is also a concern, as systemic diseases like diabetes can exacerbate periodontal conditions through altered immune responses and metabolic dysfunction [12]. Longitudinal interventional studies are essential to address these challenges and clarify whether effective periodontal treatment can mitigate systemic disease risks and vice versa [61].

**Fig. 1:**
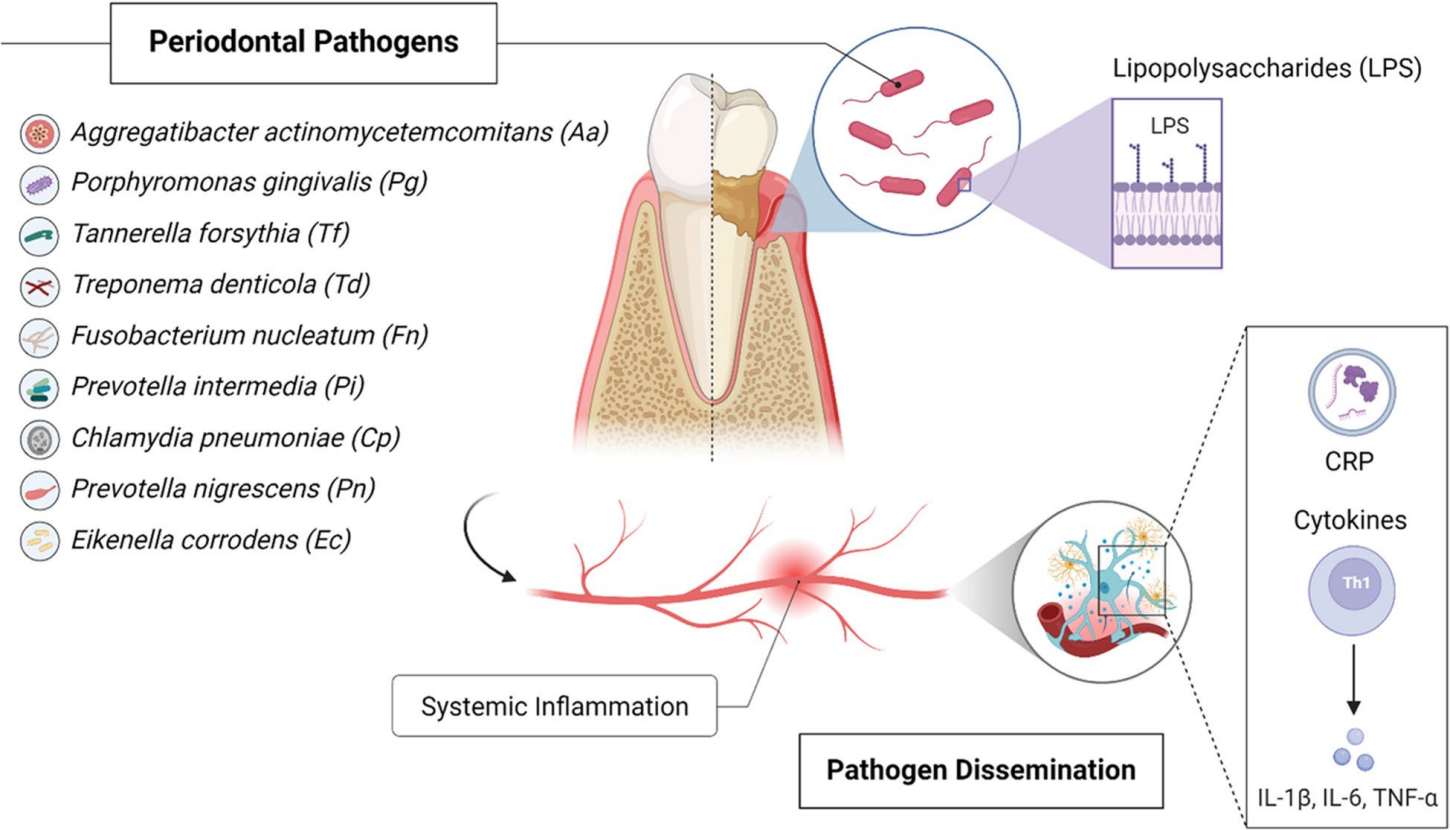
Original illustration depicting the pathways linking periodontal disease to systemic inflammation and associated health conditions. Periodontal pathogens, including *Aggregatibacter actinomycetemcomitans* (Aa), *Porphyromonas gingivalis* (Pg), *Tannerella forsythia* (Tf), *Treponema denticola* (Td), *Eubacterium nodatum* (En), *Fusobacterium nucleatum* (Fn), *Prevotella intermedia* (Pi), *Campylobacter rectus* (Cr), *Peptostreptococcus (Micromonas) micros* (Pm), *Eikenella corrodens* (Ec), *Chlamydia pneumoniae* (Cp), *Prevotella nigrescens* (Pn) and *Capnocytophaga* species (Cs), and their virulence factors, such as Lipopolysaccharides (LPS), disseminate from the infected oral cavity into the systemic circulation (pathogen dissemination). This process, along with the release of proinflammatory mediators including cytokines (Interleukin-1β [IL-1β], Interleukin-6 [IL-6], and Tumor Necrosis Factor-alpha [TNF-α]) and acute-phase proteins like C-reactive protein (CRP), contributes to systemic inflammation. This systemic inflammatory state is linked to various systemic diseases, including cardiovascular disease, diabetes mellitus, adverse pregnancy outcomes, and others discussed in this review. Immune responses involving cells like T-helper 1 (Th1) cells are also modulated

Figure 1 highlights the intricate connections between periodontal and systemic health, underscoring the critical need for early diagnosis, effective treatment, and a coordinated healthcare approach to enhance overall well-being of the patients.

#### Cardiovascular disease

**Fig. 2:**
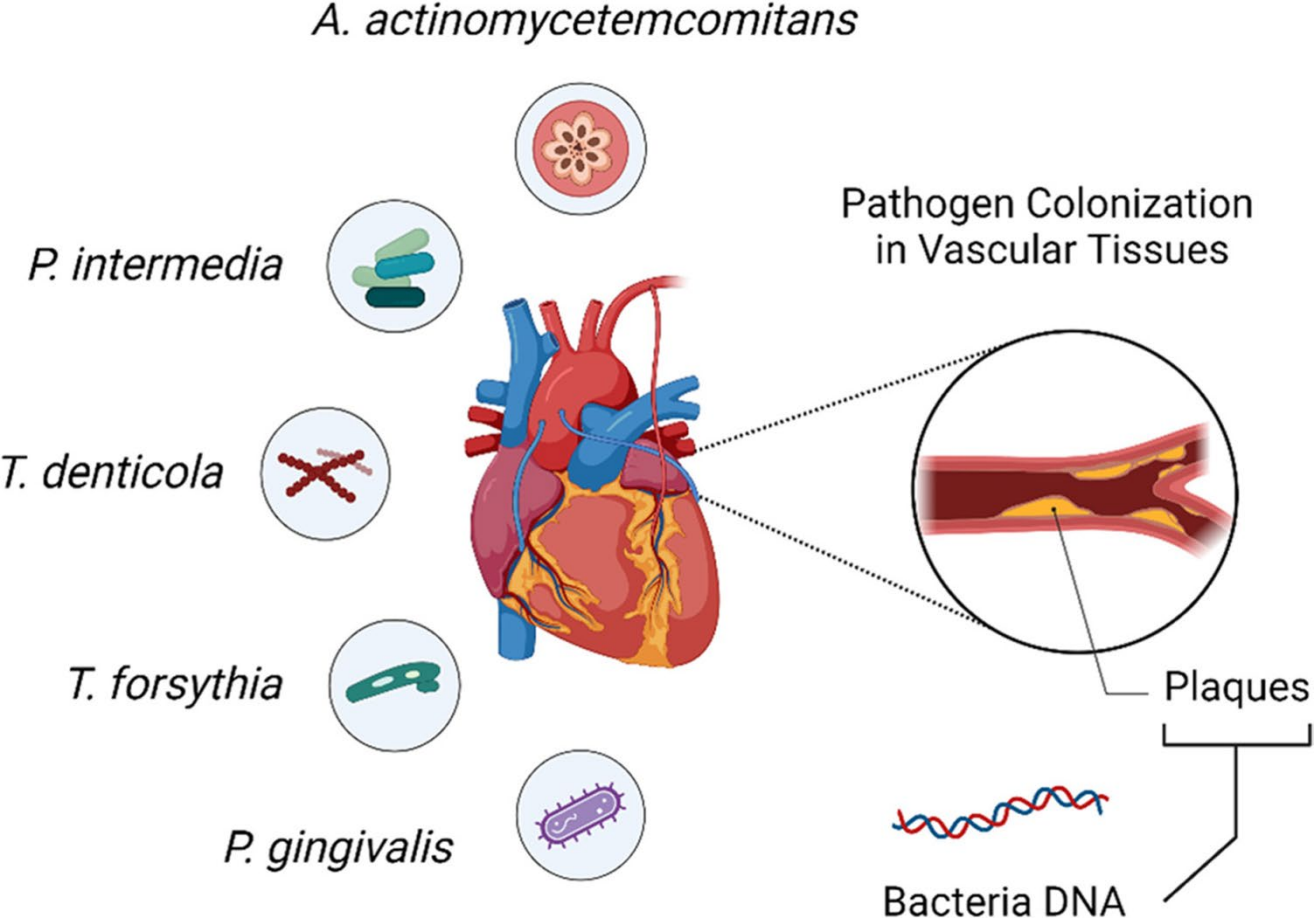
The diagram illustrates the association between periodontal pathogens, *A. actinomycetemcomitans*, *P. gingivalis*, *T. forsythia*, *T. denticola*, and *P. intermedia* and cardiovascular disease. These pathogens and their virulence factors translocate to vascular tissues, where they colonize and contribute to the formation of atherosclerotic plaques. Bacterial DNA from these pathogens has been detected in arterial plaques, linking periodontal infections to systemic vascular conditions and emphasizing the oral-systemic connection in disease etiology

Cardiovascular diseases (CVDs), encompassing disorders of the heart and blood vessels, are the leading cause of death globally, accounting for an estimated 17.9 million deaths annually. Notably, there is a significant association between periodontal disease and an increased risk of cardiovascular events. Studies have shown that individuals with periodontal disease have a higher risk of experiencing heart attacks, strokes, or other serious cardiovascular events compared to those with healthy gums [62]. Pathogens such as Aa, Pg, Tf, Td, and Pi are transferred from the periodontal region to the vascular system (Fig. 2). These pathogens may also spread into circulation during normal oral activities or dental treatments that cause bacteremia. In circulation, these bacteria may adhere to the vessel walls and participate in the pathogenesis of atherosclerosis, resulting in plaque deposition in the vessel walls. This process is mediated by the inflammatory response that is stimulated by these pathogens [19, 20, 44].

A meta-analysis of five cohort studies involving 86,092 patients revealed that individuals with periodontal disease have a 12% higher risk of developing coronary heart disease (CHD). In contrast, case–control studies with 1,423 patients demonstrated an even greater risk, with an odds ratio of 2.22 [11]. An important piece of evidence supporting this relationship is the presence of DNA from pathogens such as Pg, Aa and periodontal Tf in atheromatous plaques, which proves that oral pathogens or their microbial products travel to other parts of the body [34, 35, 40]. Animal studies further corroborate this link, showing that oral infections with Pg and Td are associated with alveolar bone loss and aortic atherosclerosis, with bacterial DNA identified in systemic tissues and organs [63, 64]. Pg possesses specific virulence factors that are implicated in the development and progression of CVD. Its major cysteine proteases, known as gingipains, play a multifaceted role. Gingipains can degrade extracellular matrix proteins and components of cell–cell junctions, potentially compromising endothelial barrier integrity and contributing to endothelial dysfunction, a key early step in atherogenesis. Furthermore, gingipains can directly activate key elements of the coagulation cascade, such as prothrombin and Factor X, while also degrading fibrinogen. This dysregulation promotes a pro-thrombotic state, increasing the risk of clot formation (thrombosis) within atherosclerotic plaques [9, 58, 65]. P. gingivalis can also hide from the innate immune system, partly due to its unique LPS structure that weakly activates Toll-like receptor-4 (TLR-4), thus potentially maintaining chronic vascular inflammation rather than triggering acute clearance [40]. Additionally, P. gingivalis can induce platelet aggregation, contributing further to thrombosis risk. This occurs through both direct and indirect mechanisms: the bacterium's outer membrane proteins and vesicles can directly bind to platelet receptors (e.g., GPIb), triggering activation and aggregation, while inflammatory mediators released in response to the infection can also secondarily activate platelets [66, 67]. These mechanisms, particularly potent in Pg compared to some other oral pathogens like Aa or Td, underscore its significant contribution to the increased risk of atherothrombotic events in individuals with periodontal disease [65]. The presence of bacterial DNA within the plaques (Fig. 2) underscores the direct role of periodontal pathogens in the development and progression of atherosclerosis. Cytokines, such as interleukin-1β (IL-1β), tumor necrosis factor-alpha (TNF-α), and acute-phase proteins, including CRP, which are secreted during periodontitis, enhance systemic inflammation, which in turn aggravates endothelial dysfunction and plaque formation. This systemic inflammation not only worsens atherosclerosis but also increases the risk of myocardial infarction and stroke [11].

#### Diabetes mellitus

**Fig. 3:**
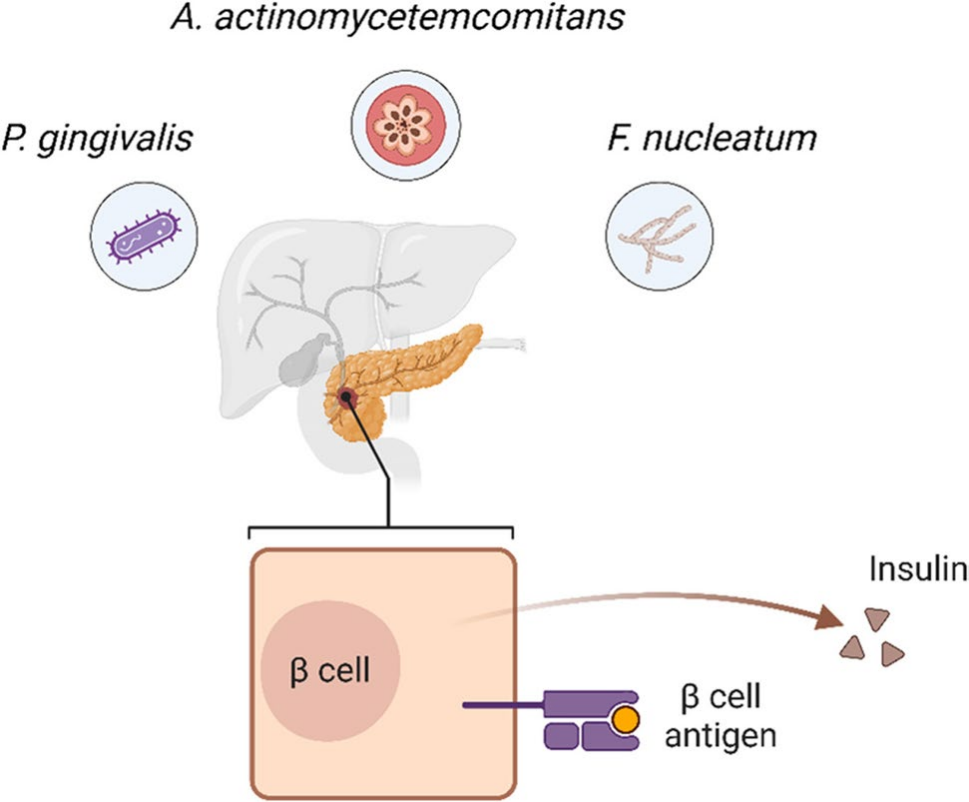
The diagram shows the impact of periodontal pathogens *Aggregatibacter actinomycetemcomitans* (Aa), *Porphyromonas gingivalis* (Pg), and *Fusobacterium nucleatum* (Fn) on β-cell function in the pancreas. These pathogens contribute to inflammation and immune-mediated damage, potentially impairing β-cell antigen presentation and insulin production, and ultimately exacerbating insulin resistance and the development or progression of diabetes mellitus

Diabetes mellitus is a chronic disease of glucose metabolism in which there is an increase in blood glucose due to insulin deficiency (type 1 diabetes), insulin resistance (type 2 diabetes) or both [68]. This condition can be seen in adults, teenagers, children, and even infants. Diabetes mellitus and periodontitis are related to each other in a way that one complicates the other (Fig. 3). This bidirectional link is greatly affected by the interactions between periodontal pathogens and insulin-producing pancreatic β cells [69]. Periodontitis is a chronic inflammatory disease that leads to inflammation of the periodontal tissues and other oral tissues and is associated with increased levels of cytokines and acute-phase proteins. Periodontal diseases and type 2 diabetes share significant inflammatory components. Systemic infections, like the common cold or influenza, elevate systemic inflammation, thereby increasing insulin resistance and complicating blood glucose management [70]. Chronic periodontal diseases can further exacerbate insulin resistance and impair glycemic control, whereas periodontal treatment that reduces inflammation may improve insulin sensitivity. This systemic inflammation can interfere with insulin signaling, contributing to insulin resistance and poor glycemic control [12, 71].

Several periodontal pathogens, including Pg, Fn and Aa, may play critical roles in this bidirectional association. These pathogens cause inflammation and alter the immune response which may affect beta cell function and exacerbate systemic metabolic dysregulation [68]. Figure 3 highlights the role of β cells and insulin production. Conversely, hyperglycemia in diabetes impairs immune function, making it harder to control periodontal infections, leading to more severe periodontal destruction [52]. Early studies have suggested higher bacterial proportions in the periodontal pockets of diabetic patients [72]. However, later culture-based studies found minimal differences between periodontally diseased sites in diabetic and non-diabetic subjects [73]. Consequently, research has shifted to examining differences in the immunoinflammatory response to bacteria between diabetic and non-diabetic individuals [74]. In many diabetics, the function of key immune cells is altered [75]. Neutrophil adherence, chemotaxis, and phagocytosis are often impaired, hindering bacterial destruction in periodontal pockets [76]. Beyond neutrophils, macrophage function is also compromised; this includes reduced phagocytic capacity, which further limits bacterial clearance, and alterations in their polarization and ability to promote the resolution of inflammation, contributing to persistent rather than resolving inflammatory states. Additionally, diabetics exhibit upregulated immunoinflammatory responses, with monocytes and macrophages producing elevated levels of proinflammatory cytokines like TNF-α in response to periodontal pathogens, potentially enhancing tissue destruction [77]. Diabetic individuals are three times more likely to develop periodontitis than non-diabetic individuals, with studies reporting a 58% prevalence of periodontitis in patients with type 1 diabetes compared with only 15% in non-diabetic controls [52, 78]. Diabetics often experience impaired wound healing [79]. In high-glucose environments, periodontal fibroblasts, the primary reparative cells, function suboptimally [80]. Additionally, collagen produced by the fibroblasts is rapidly degraded by matrix metalloproteinases, which are elevated in diabetes [81]. Consequently, chronic hyperglycemia may alter periodontal wound healing, leading to increased bone and periodontal attachment loss. Diabetics, particularly those with poor glycemic control, accumulate high levels of advanced glycation end products (AGEs) in tissues, including the periodontium. AGEs are linked to various diabetic complications by inducing significant changes in cells and extracellular matrix components [74]. Crucially, AGEs exert many of their pro-inflammatory effects by binding to their cell surface receptor, RAGE (Receptor for Advanced Glycation End products). This AGE-RAGE signaling axis is upregulated in diabetes and acts as a potent amplifier of inflammation. Activation of RAGE on immune cells (like macrophages) and resident periodontal cells triggers intracellular signaling cascades that lead to increased production of pro-inflammatory cytokines (e.g., TNF-α, IL-6) and MMPs, thereby exacerbating the inflammatory response, promoting tissue destruction, and contributing to complications like abnormal endothelial cell function, altered capillary growth, and vessel proliferation within the periodontium [74, 81].

This relationship is even more important in children and adolescents with type 1 diabetes, as they are more likely to develop gingivitis, which is seen in 21% of the cases, and periodontitis in 6% of cases as compared to children and adolescents who do not have diabetes [82]. In addition, patients with type 1 diabetes have been found to have worse periodontal disease parameters when they have had the disease for more than five years [83]. Interestingly, treatment of periodontal infections enhances glycemic control in patients with diabetes, thus underlining the importance of concurrent management of both diseases [52]. Evidence suggests that diabetics with periodontitis may be at higher risk for poor glycemic control and that periodontal treatment reducing oral inflammation might improve glycemic control. However, this evidence is not conclusive. Large, randomized, controlled trials are needed to strengthen the available evidence. Inflammation is a common link between periodontal diseases and diabetes, and further research is needed to elucidate how inflammatory periodontal diseases impact insulin resistance, glycemic control, and the risk of other diabetic complications.

#### Adverse pregnancy outcomes

**Fig. 4:**
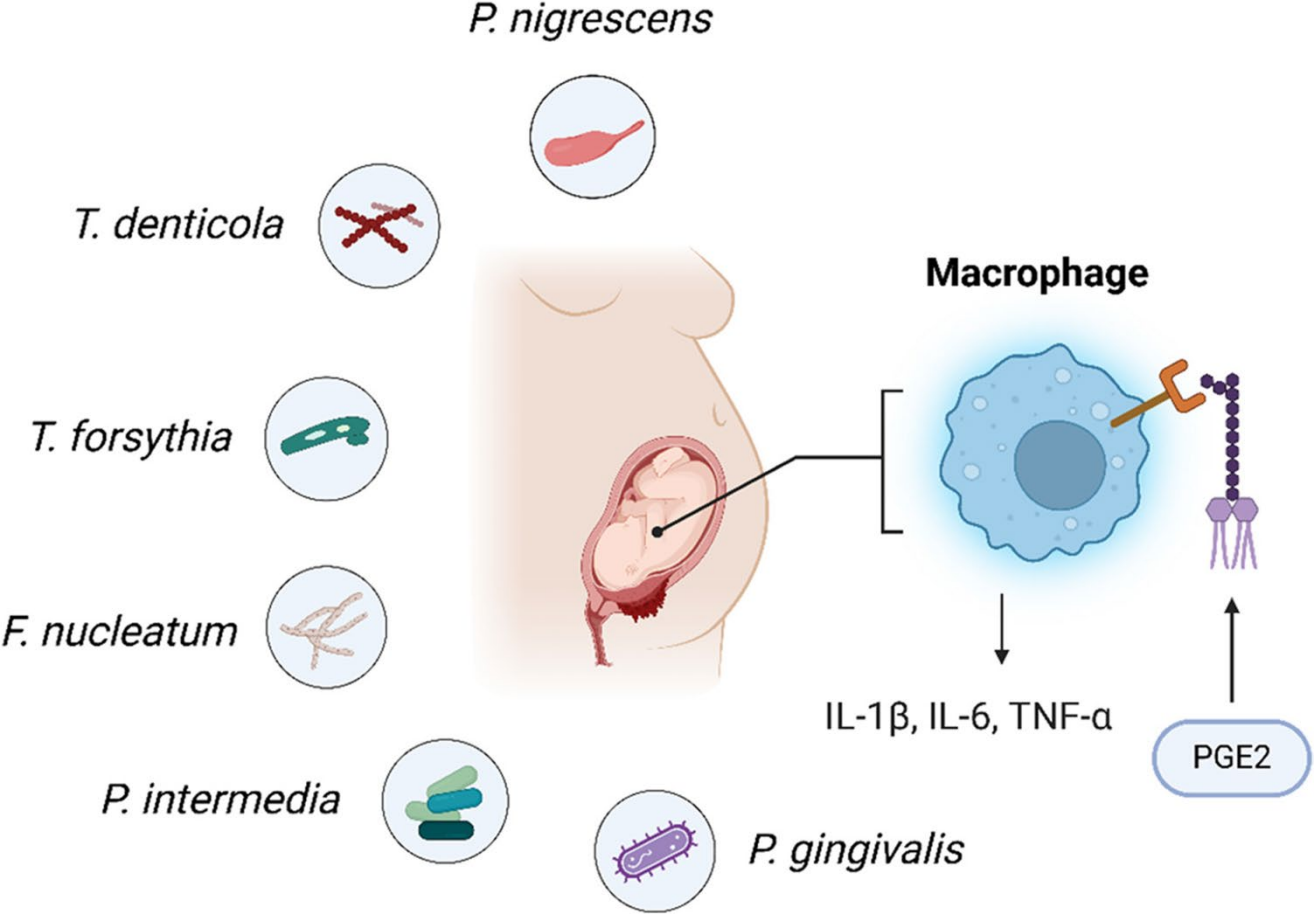
Illustration depicting the role of periodontal pathogens (*P. gingivalis*, *F. nucleatum*, *T. forsythia*, *T. denticola*, *P. intermedia*, and *P. nigrescens*) in adverse pregnancy outcomes. These pathogens translocate to the placenta, activate macrophages, and release proinflammatory mediators, such as IL-1β, IL-6, TNF-α, and PGE2. The inflammatory response disrupts the maternal–fetal environment, contributing to complications, such as preterm birth, low birth weight, and preeclampsia

Pregnancy alters the hormonal status of females and hence increases the risk of periodontal diseases including gingivitis and periodontitis, compared to non-pregnant women [84]. This increased susceptibility poses a higher risk of periodontal pathogens affecting pregnancy outcomes. It is thought that some periodontal pathogens or their microbial products may be released into the bloodstream and then transported to the placenta; these include Fn, Pg, Pi, Tf, Td, and Pn (Fig. 4). As shown in Fig. 4, these pathogens could invade maternal immune cells such as macrophages in the placental environment. This contact stimulates synthesis of proinflammatory cytokines such as Interleukin 1β (IL-1β), Interleukin 6 (IL-6), Tumor Necrosis Factor alpha (TNF-α), and Prostaglandin E2 (PGE2) [85]. Pathogens and consequent inflammation have been associated with adverse pregnancy outcomes such as preterm birth, low birth weight, preeclampsia, miscarriage and stillbirth [14].

The release of proinflammatory mediators such as prostaglandins and interleukin- 6 during periodontal inflammation may negatively affect the maternal–fetal environment, thus increasing the chances of conditions such as intrauterine growth restriction, neonatal sepsis and premature rupture of membranes [14, 45]. These findings highlight the critical importance of maintaining periodontal health during pregnancy to reduce the risk of complications and ensure favorable outcomes for both the mother and infant. Evidence indicates that certain periodontal treatment interventions may reduce the risk of preterm birth and low birth weight. Future research should aim to optimize these intervention strategies and determine the best timing during pregnancy to enhance healthcare for pregnant women [86].

#### Alzheimer’s disease

**Fig. 5:**
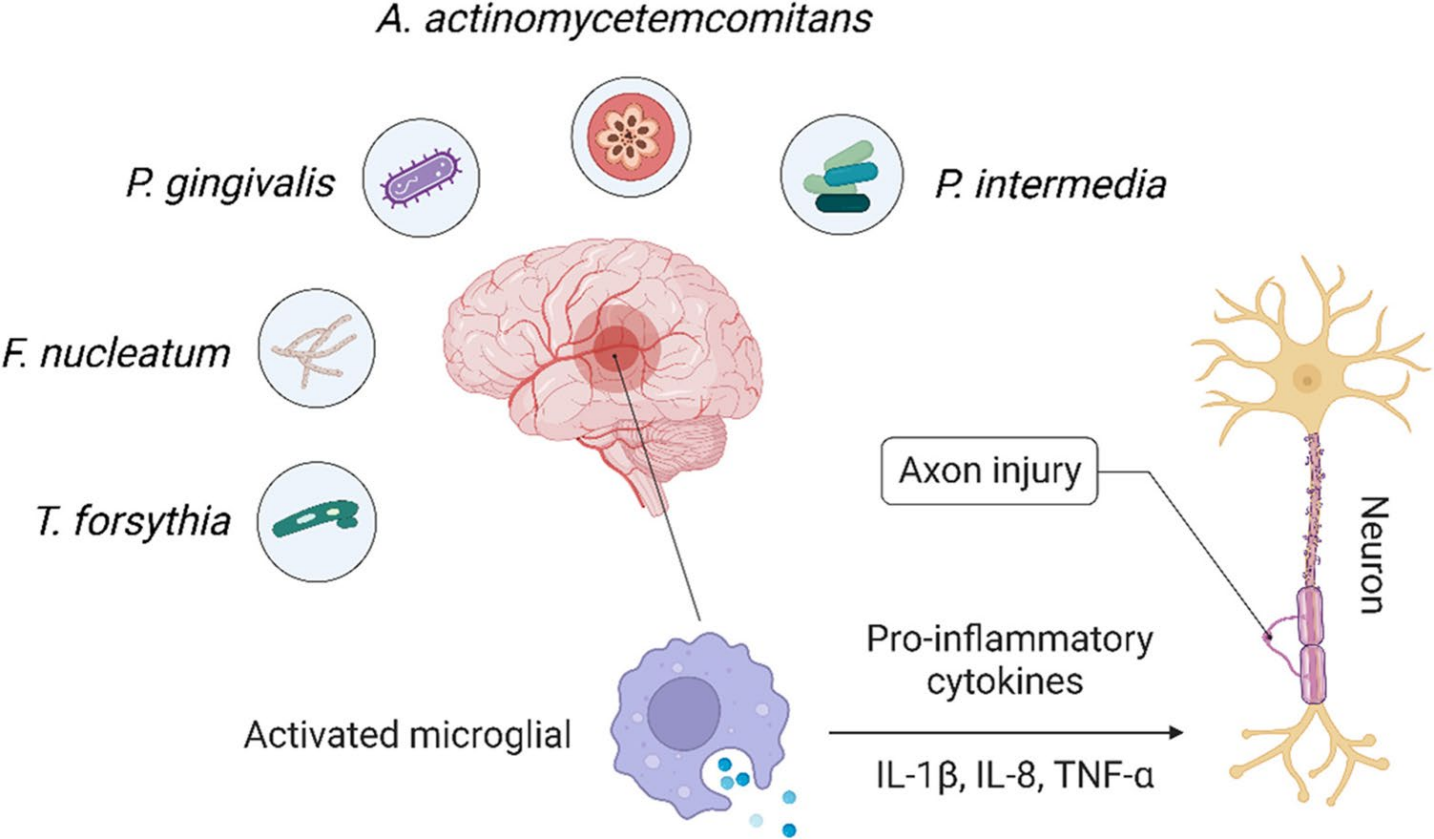
Illustration demonstrating the impact of periodontal pathogens (*A. actinomycetemcomitans*, *P. gingivalis*, *T. forsythia*, *F. nucleatum*, and *P. intermedia*) on neuroinflammation and neuronal damage. These pathogens or their microbial products such as LPS activate microglial cells in the brain, leading to the release of proinflammatory cytokines (IL-1β, IL-8, and TNF-α). The resulting inflammation contributes to axon injury, highlighting the role of periodontal infections in the pathogenesis of neurodegenerative diseases, such as Alzheimer’s disease

Alzheimer's disease (AD) is a chronic and irreversible disease characterized by the gradual loss of cognitive functions such as memory, thinking, language and learning ability and resulting ultimately in death [87]. The cognitive deficits associated with AD are primarily linked to the accumulation of synaptotoxic β-amyloid plaques and hyperphosphorylated tau proteins in brain regions responsible for advanced cognitive functions [87, 88]. There is now evidence of crosstalk between AD and periodontal diseases, in which systemic inflammation and microbial transmission from the oral cavity to the brain have been postulated to induce AD. As shown in Fig. 5, several periodontal pathogens, including Pg, Fn, Aa, Tf, and Pi, which are involved in this process, can induce neuroinflammation and AD via various mechanisms. Chronic periodontitis elevates circulating proinflammatory cytokines, which stimulate brain microglia. Activated microglia then secrete additional proinflammatory cytokines, such as IL-1β, IL-8, and TNF-α, which can cross the blood–brain barrier and exacerbate neuroinflammation associated with Alzheimer's disease [89, 90]. Lipopolysaccharides (LPS) from *P. gingivalis* and *Treponema denticola* have been detected in postmortem Alzheimer’s disease brains, highlighting their potential role in brain inflammation [91]. Additionally, these pathogens produce virulent factors such as gingipains, which degrade brain tissue and promote the formation of amyloid-beta plaques, a hallmark of AD [91].

Further evidence has also been obtained from the analysis of periodontal pathogens such as *T. denticola, P. gingivalis*, and *C. pneumoniae* in human and animal brains demonstrating their potential to cross the blood–brain barrier and cause neurodegeneration [92–95]. Levels of antibodies against Aa, Pg, Tf, Fn, and Pi were higher in elderly patients with AD than in controls, strengthening the relationship between periodontal infection and cognitive impairment [90, 96]. The results of these studies suggest that maintaining periodontal health could play a crucial role in preserving cognitive function and preventing Alzheimer's disease in the elderly. Chronic periodontitis, a common inflammatory condition, has been shown to elevate levels of circulating proinflammatory cytokines [97]. These cytokines can activate microglia in the brain, leading to increased secretion of additional proinflammatory cytokines such as IL-1β, IL-8, and TNF-α. These cytokines can cross the blood–brain barrier, exacerbating neuroinflammation, which is a key factor in the pathogenesis of Alzheimer's disease [98]. Therefore, effective periodontal treatment and management may help mitigate systemic inflammation and its detrimental effects on the brain, highlighting the importance of oral health in the overall well-being and cognitive health of the elderly population. Further research is needed to fully understand the mechanisms linking periodontal health and cognitive function, and to develop targeted interventions for preventing neurodegenerative diseases.

#### Oral and colorectal cancer

**Fig. 6:**
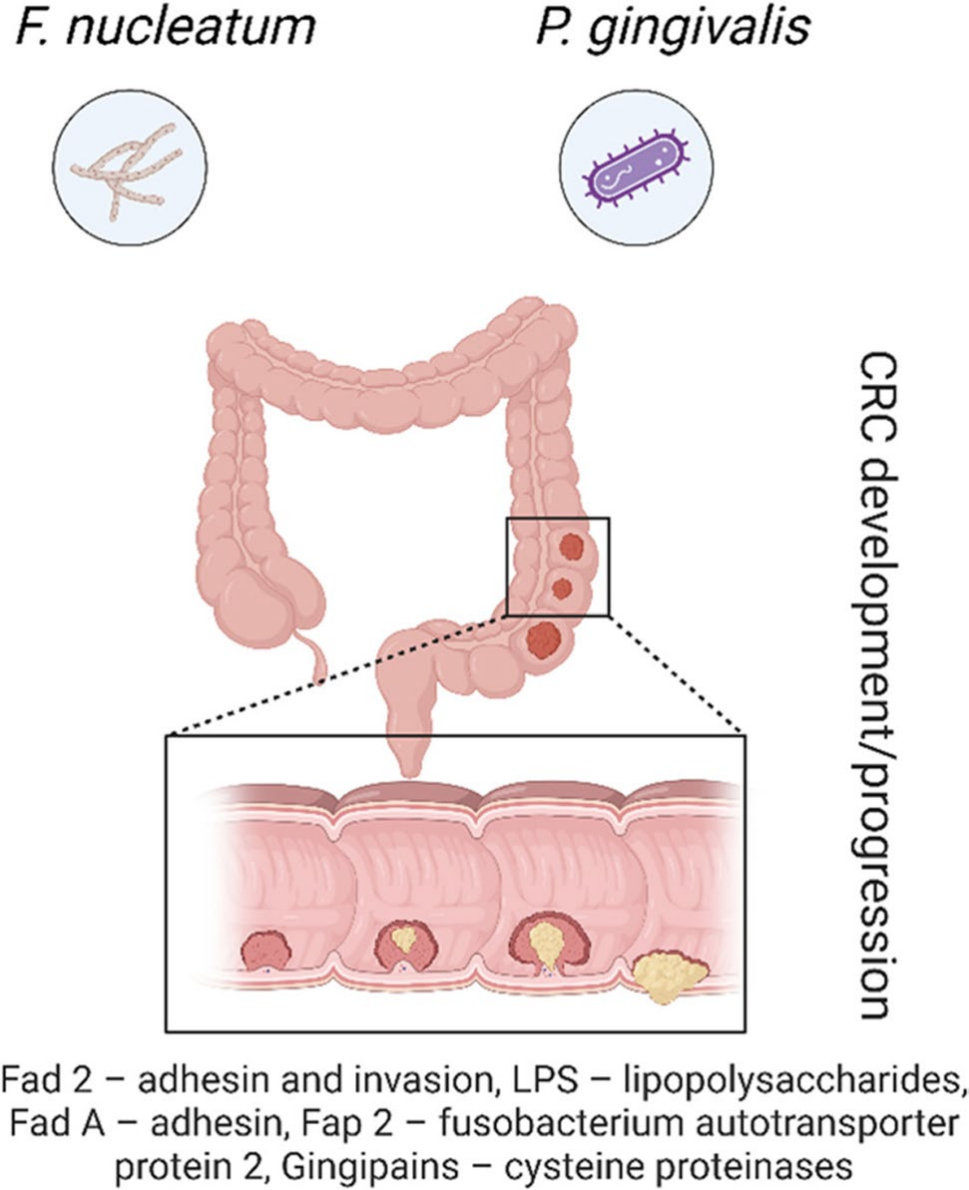
Diagram illustrates the role of periodontal pathogens (*F. nucleatum* and *P. gingivalis*) in the development and progression of colorectal cancer (CRC). These pathogens contribute to CRC through mechanisms such as adhesion and invasion (Fad2 and FadA), the production of LPS, fusobacterium autotransporter protein 2 (Fap2), and gingipains (cysteine proteinases). The lower inset highlights tumor development in the colon epithelial lining, emphasizing the systemic impact of periodontal pathogens on CRC pathogenesis

Periodontal diseases have been associated with an increased risk of certain cancers, including oral squamous cell carcinoma and colorectal cancer (CRC) [99, 100]. Two major periodontal pathogens, Pg and Fn, have been linked with CRC. As shown in Fig. 6, these bacteria are located in the large intestine where CRC occurs. *Fn* in particular has been associated with CRC, and a specific clade of Fn is thought to cause CRC [101]. Pg may enhance the invasive and metastatic potential of oral squamous cells by increasing the levels of MMP-9, which is involved in tissue degradation and cancer progression. [102]. A meta-analysis involving 3,183 subjects showed that patients with periodontal disease are at a higher risk of developing oral cancer thus underlining the importance of oral health in cancer risk [103]. A large-scale study of one million randomly selected insurance patients in Taiwan also showed that patients with periodontitis had an increased risk of colon cancer compared to those with simple gingivitis thus supporting the concept of periodontal inflammation as a risk factor for systemic carcinogenesis [104]. The association between periodontal disease and other cancers including pancreatic, head and neck and lung cancers supports the notion that periodontal health can be beneficial for reducing cancer risk [105]. These results demonstrate the systemic effects of periodontal pathogens and the need for overall oral health to prevent cancer.

In the magnified view depicted in Fig. 6, *Fusobacterium nucleatum* (Fn) is shown interacting with the intestinal epithelium, potentially contributing to tumor formation by stimulating a proinflammatory microenvironment and modulating immune responses. It promotes tumor growth by attracting myeloid-derived suppressor cells, which suppress the immune response to tumors. The presence of Fn in colorectal tumors further supports its role as a microbial driver of cancer progression [17]. Figure 6 also highlights virulence factors associated with Fn and Pg, including Fad 2 (adhesin and invasion), LPS, Fad A (adhesin), Fap 2 (fusobacterium autotransporter protein 2), and gingipains (cysteine proteinases), all of which could contribute to the development and progression of CRC. Individuals with periodontal diseases have an increased risk of gastrointestinal cancer compared to those without periodontal diseases [106]. Specifically, periodontal diseases significantly raise the risk of esophageal cancer by 39% gastric cancer by 13%, colorectal cancer by 21%, pancreatic cancer by 35%, and liver cancer by 9% [107]. The risk is further increased by periodontitis, gingivitis, and combined periodontitis/gingivitis. Patients with severe periodontal diseases have a significantly higher risk of gastrointestinal cancer [108]. Thus, periodontal diseases, particularly severe periodontitis, elevate the risk of gastrointestinal cancer. Preventive and management interventions for periodontal diseases may reduce this risk [109].

#### Rheumatoid arthritis

**Fig. 7:**
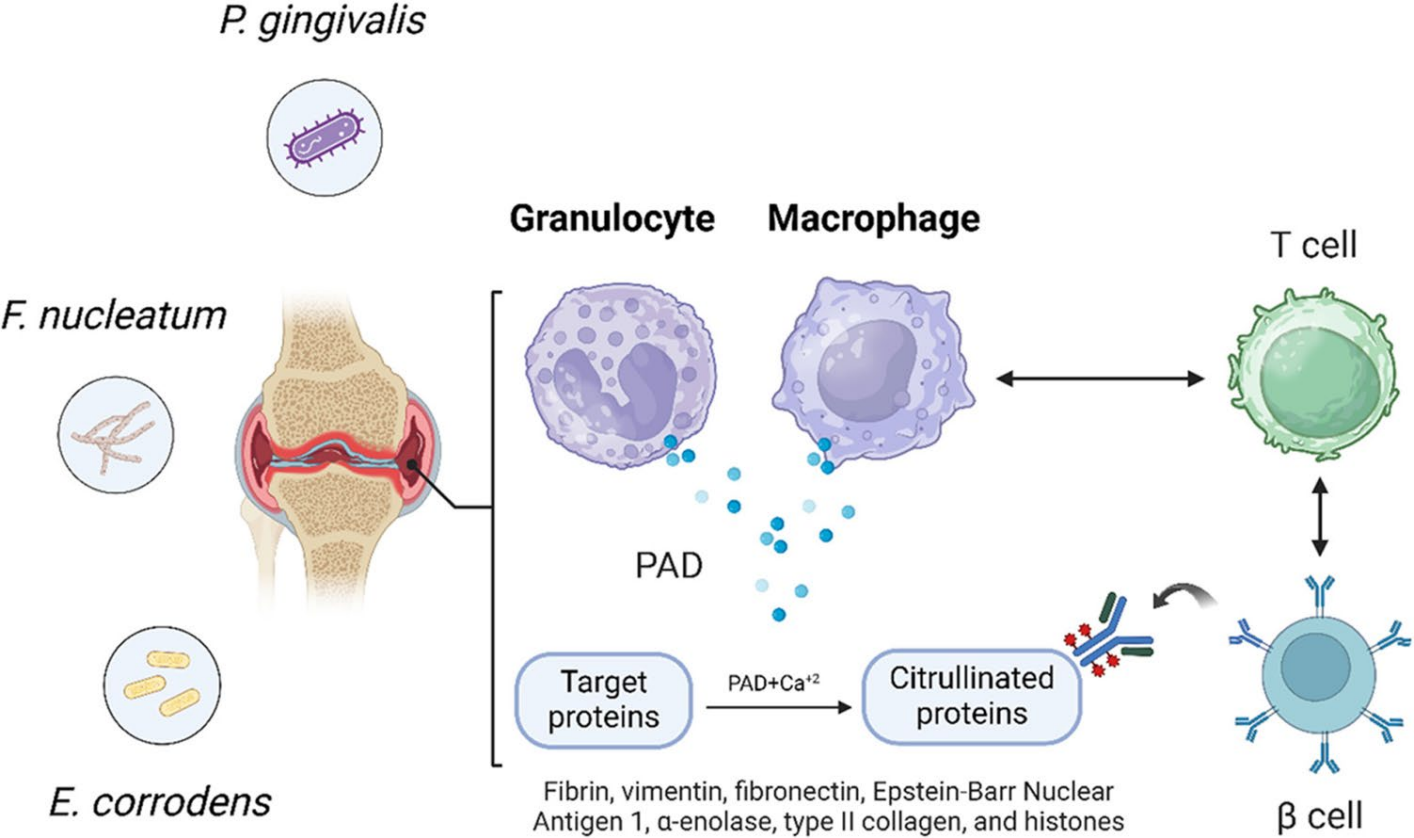
Diagram illustrates the role of periodontal pathogens (*P. gingivalis*, *F. nucleatum*, and *E. corrodens*) in the pathogenesis of rheumatoid arthritis (RA). These pathogens contribute to protein citrullination through the activity of peptidylarginine deiminase (PAD), leading to the conversion of target proteins (e.g., fibrin, vimentin, and type II collagen) into citrullinated forms. These citrullinated proteins trigger autoimmune responses by activating T and B cells, resulting in the production of autoantibodies and perpetuation of joint inflammation in RA

Periodontal disease has been linked to autoimmune conditions, such as rheumatoid arthritis (RA), because both conditions are fueled by inflammatory pathways and feature microbial dysbiosis, which is central to their pathogenesis [110]. A specific enzyme, peptidylarginine deiminase (PAD), produced by Pg, converts proteins (fibrin, vimentin, fibronectin, Epstein-Barr Nuclear Antigen 1, α-enolase, type II collagen, and histones) into citrullinated antigens in the presence of calcium ions (Ca^2^⁺) [111]. These modified proteins give rise to autoimmune responses when they are recognized by antibodies produced by B cells after interaction with T cells [112]. Fn and Ec worsen the condition by inducing immune dysregulation and releasing proinflammatory cytokines, which worsen joint pain and swelling associated with RA. This chronic inflammatory state worsens RA symptoms and creates a vicious cycle in which people with RA are more likely to develop severe periodontitis because of their dysregulated immune system [113]. These processes are visually represented in Fig. 7, which shows an inflamed joint depicting how periodontal pathogens and immune mechanisms could jointly contribute to autoimmune inflammation [114].

Clinical research has indicated that managing periodontal diseases can help reduce inflammation throughout the body, thus improving the treatment of RA. Studies on the effects of periodontal therapy on RA have revealed that autoimmune inflammation can be reduced by treatment, emphasizing the need to manage oral health when treating autoimmune diseases such as RA [115]. This underscores the need for integrated care approaches to address both periodontal and systemic autoimmune health issues. Controlling periodontal disease through non-surgical periodontal treatment can significantly reduce the severity of rheumatoid arthritis [116]. This finding strongly supports the hypothesis of a pathogenic link between these two chronic inflammatory conditions. By mitigating periodontal inflammation, non-surgical treatments may decrease systemic inflammatory markers, which in turn can alleviate RA symptoms. This underscores the importance of integrated healthcare approaches that address both oral and systemic health to improve patient outcomes. Further research is warranted to elucidate the underlying mechanisms and to optimize treatment protocols for managing these interconnected diseases.

#### Respiratory tract infections and pneumonia

**Fig. 8:**
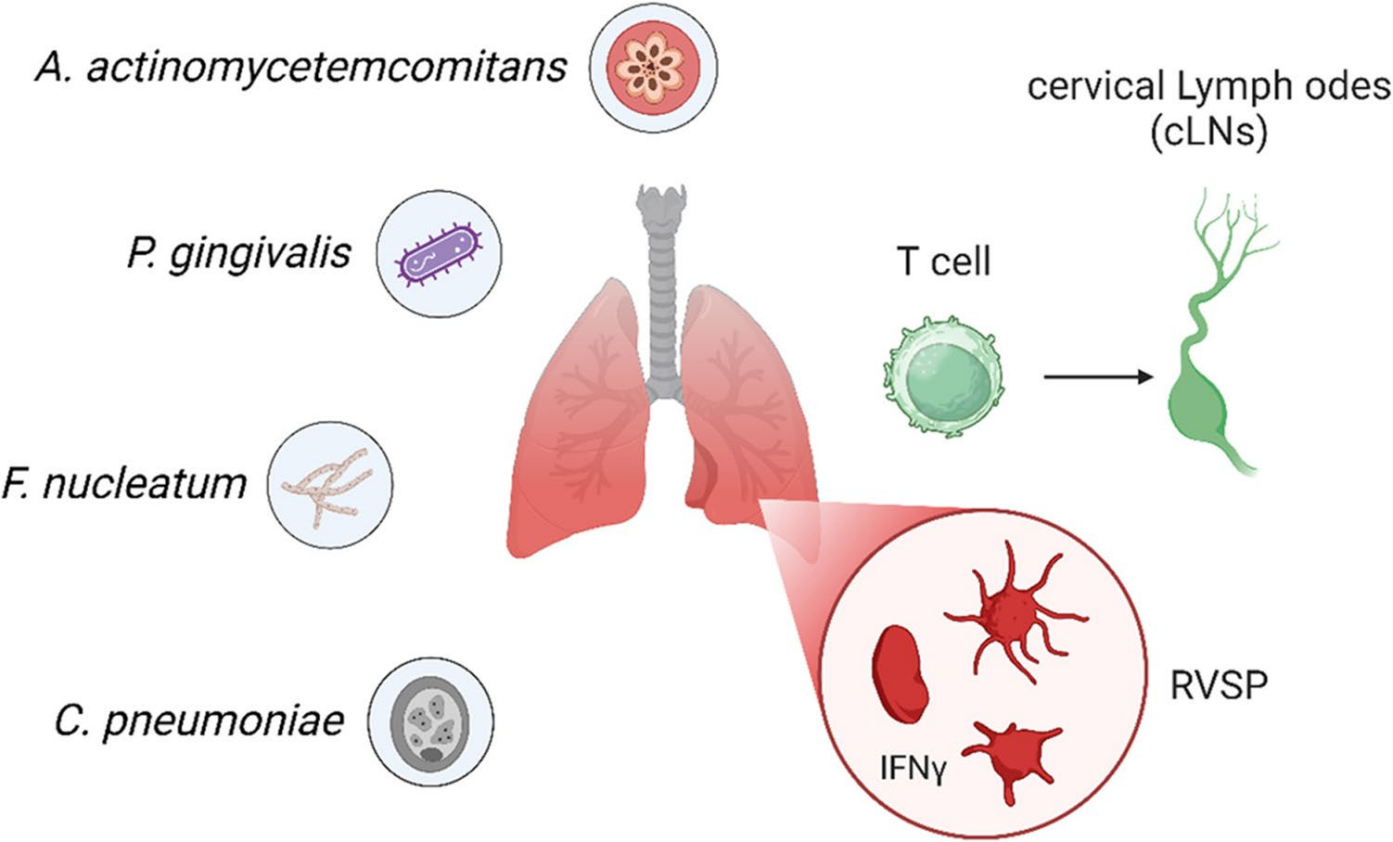
Mechanistic illustration of periodontitis-mediated aggravation of pulmonary hypertension (PH) via the oral-lung axis. Periodontal pathogens, including *A. actinomycetemcomitans*, *P. gingivalis*, *F. nucleatum*, and *C. pneumoniae*, contribute to PH through two mechanisms: (1) expansion of IFNγ + T cells in cervical lymph nodes (cLNs) triggered by oral pathogens, and (2) translocation of pathogens to the lungs, leading to increased infiltration of IFNγ + T cells in pulmonary tissues. This process is associated with an elevated right ventricular systolic pressure (RVSP), which highlights the interplay between oral and lung health in the progression of systemic diseases

Periodontal disease is associated with respiratory tract infections, including pneumonia, where the risk is especially high in certain groups of people, including the elderly, hospitalized patients, and patients undergoing orotracheal intubation [117]. As shown in Fig. 8, the following oral pathogens are involved in this connection: Pg, Fn, Aa, and Cp. Other microorganisms including Actinomyces israelii, Capnocytophaga spp., Eikenella corrodens, Fusobacterium necrophorum, Prevotella intermedia, Streptococcus constellatus can be released into the lower respiratory tract, where they may adhere to the tissue and cause inflammation and colonization that resulting in infections such as pneumonia [118–120]. Studies have shown that respiratory pathogens isolated from dental plaque and bronchoalveolar lavage fluid in intensive care patients who are genetically identical, reinforcing the role of dental plaque as a reservoir for respiratory pathogens [121]. Research has also indicated that patients with periodontitis are three times more likely to develop nosocomial pneumonia than those without periodontitis [122].

Oral infection by periodontal pathogens also worsens lung tissue injury and respiratory complications through the inflammatory response [123]. When cytokines such as IL-6 and TNF-α and enzymes that are released during periodontal inflammation are produced, this enhances the systemic inflammatory response and the risk of developing pneumonia [124, 125]. Experimental models have shown that P. gingivalis infection leads to chronic inflammation and cytokine release in the lungs as well as *F. nucleatum* and *F. necrophorum* which have been associated with Lemierre’s syndrome and whose infection starts as pharyngitis and then spreads to the respiratory tract infection [119, 120]. However, C. pneumoniae, which is a classical respiratory tract pathogen, was also isolated in the oral cavity raising the possibility of its migration from the oral cavity to the lower airways and subsequent spread to other organs and tissues with systemic effects including atherosclerosis [126].

A working model has been proposed that periodontitis aggravates pulmonary hypertension (PH) through an oral-lung axis by facilitating the translocation of *P. zoogleoformans* to the lungs, inducing IFNγ + T cell expansion in cervical lymph nodes (cLNs) and lungs, leading to heightened inflammation and elevated right ventricular systolic pressure (RVSP) [127].

Some clinical studies have focused on the effect of good oral hygiene and periodontal treatment on preventing respiratory infections, with special emphasis on high-risk groups [128]. In a mouse model, Pg was detected in various systemic organs, underlining the relationship between oral pathogens and respiratory and systemic diseases [63]. Surprisingly, even edentulous patients were found to harbor high levels of Aa, Pg and T. forsythia which indicates that the oral cavity with or without teeth is a suitable place for growth of pathogenic bacteria [124, 129]. These findings support the concept of a periodontal-respiratory disease relationship and the importance of dental prevention in reducing respiratory diseases.

In conclusion, the systemic effects of periodontal disease highlight the profound interconnectivity between oral and systemic health. Inflammatory and microbial mechanisms in the oral cavity influence conditions such as cardiovascular disease, diabetes mellitus, adverse pregnancy outcomes, Alzheimer's disease, and cancer. These connections underscore the importance of integrated oral healthcare and present opportunities for multiprofessional approaches in preventing and treating systemic diseases caused by periodontal diseases. Effective periodontal health maintenance measures can positively impact overall patient health by reducing systemic inflammation and its associated risks. This integrated approach emphasizes the need for collaboration among dental and medical professionals to enhance patient outcomes and promote holistic health. Further research is essential to fully understand these interconnections and to develop comprehensive strategies for managing both oral and systemic health.

## Key biomarkers for diagnostics

Biomarkers are indicators used for the diagnosis of disease and its progression, as well as providing non-invasive and accurate methods for diagnosing the association between periodontal disease and systemic diseases. Saliva, blood, and GCF have become popular biological samples for assessing inflammatory and microbial markers. The main biomarkers detected in saliva, blood, and GCF are presented in Table 1, along with the systemic effects of these biomarkers and their relevance to the association between periodontal diseases and other systemic diseases.

**Table 1:** Biomarkers in Saliva, Blood, and GCF and Their Systemic Implications

Biological sample	Biomarker	Pathogen	Systemic implication	Clinical significance	References
Saliva	Interleukin-1β (IL-1β)	Aa, Pi, Td, Tf, Pg, Fn	Cardiovascular disease, Diabetes, Neurodegeneration	Indicator of inflammation; elevated in periodontitis and systemic inflammatory conditions	[136]
	Tumor Necrosis Factor-α (TNF-α)	Aa, Pi, Td, Tf, Pg, Fn, Pn	Cardiovascular disease, Diabetes, Adverse Pregnancy Outcomes	Associated with systemic inflammation, endothelial dysfunction, and insulin resistance	[132]
	Matrix Metalloproteinases (MMPs)	Aa, Pg, Cp,Pg, Fn, Ec	Bone loss, Respiratory infections	Reflective of extracellular matrix degradation in periodontitis and systemic tissue destruction	[131]
	Microbial DNA (e.g., P. gingivalis, F. nucleatum)	Aa, Pi, Td, Tf, Pg, Fn, Pn	Cardiovascular disease, Colorectal cancer, Adverse Pregnancy Outcomes	Indicates periodontal pathogens’ translocation and their systemic impacts	[15]
	Amyloid Beta (Aβ)	Aa, Pi, Tf, Pg, Fn	Neurodegenerative disorders (Alzheimer’s disease)	Early biomarkers for Alzheimer’s pathology linked to chronic inflammation	[137]
Blood	C-Reactive Protein (CRP)	Aa, Pi, Td, Tf, Pg, Fn, Cp	Cardiovascular disease, Respiratory infections, Diabetes	Marker of systemic inflammation; elevated in periodontitis and systemic conditions	[20]
	Interleukin-6 (IL-6)	Aa, Pi, Td, Tf, Pg, Fn, Pn	Cardiovascular disease, Diabetes, Adverse Pregnancy Outcomes	Promotes systemic inflammation; linked to insulin resistance and fetal growth restriction	[132]
	Advanced Glycation End-Products (AGEs)	Aa, Pi, Pg, Fn, Tf	Diabetes, Neurodegenerative disorders	Correlated with glycemic control and neuroinflammation	[53]
	Lipopolysaccharides (LPS)	Aa, Pi, Td, Tf, Pg, Fn	Cardiovascular disease, Colorectal cancer, Neurodegenerative disorders	Activates systemic inflammation via TLR4 signaling; linked to endothelial dysfunction and cancer	[138]
	Procalcitonin	Aa, Pg, Cp, Fn	Respiratory infections	Marker of bacterial infections; elevated in periodontitis-induced pulmonary infections	[129]
GCF	RANKL (Receptor Activator of Nuclear Factor Kappa-B Ligand)	Pg, Fn, Ec	Bone loss, Systemic osteoporosis	Indicative of bone resorption activity and systemic bone health	[135]
	Osteoprotegerin (OPG)	Pg, Fn, Ec	Bone health	Regulates bone metabolism; imbalance with RANKL indicates heightened bone loss	[10]
	Matrix Metalloproteinases (MMPs)	Pg, Fn, Ec	Bone and tissue degradation	Markers of periodontal and systemic tissue destruction	[131]
	Fetal Fibronectin (fFN)	Pn, Td, Tf, Fn, Pi, Pg	Adverse Pregnancy Outcomes	Marker of placental inflammation and risk of preterm birth	[139]
	Tumor Biomarkers (e.g., CEA, CA19-9)	Fn, Pg	Colorectal cancer	Indicates systemic effects of F. nucleatum on tumorigenesis	[140]

* Periodontal pathogens, including Aggregatibacter actinomycetemcomitans (Aa), Porphyromonas gingivalis (Pg), Tannerella forsythia (Tf), Treponema denticola (Td), Eubacterium nodatum (En), Fusobacterium nucleatum/periodonticum (Fn), Prevotella intermedia (Pi), Campylobacter rectus (Cr), Peptostreptococcus (Micromonas) micros (Pm), Eikenella corrodens (Ec), Prevotella nigrescens (Pn) and Capnocytophaga species (gingavalis, ochracea, sputigena) (Cs)

***Saliva:*** Saliva is used often as a diagnostic fluid because it is easily accessible and contains both systemic and local markers. The proinflammatory cytokines and protease IL-1β, TNF-α, and matrix metalloproteinases (MMPs) are elevated in the saliva of periodontitis patients [130, 131]. Microbial DNA from periodontal pathogens such as *P. gingivalis* and *F. nucleatum* can also be identified in saliva, providing insights into systemic dissemination [15]. The analysis of salivary biomarkers is a non-invasive method for monitoring periodontal disease activity and has the potential for diagnosing other systemic diseases.

***Blood:*** Systemic inflammatory markers such as CRP, IL-6, and advanced glycation end products (AGEs) are elevated in patients with severe periodontitis [20, 132]. Periodontal treatment reduces these systemic biomarkers, further supporting their role in periodontal-systemic interactions [61]. These biomarkers can be assessed through blood tests, which provide the clinician with details on the level of inflammation present in the body due to periodontitis.

***Gingival Crevicular Fluid (GCF):*** GCF is a local secretion containing a large number of inflammatory mediators and microbial products [133, 134]. Elevated levels of receptor activator of nuclear factor kappa-Β ligand (RANKL), osteoprotegerin (OPG), and matrix metalloproteinases (MMP-8, MMP-9) in GCF are indicative of periodontitis and reflect increased bone resorption, which is also relevant in the context of systemic bone diseases such as osteoporosis [10, 135]. RANKL enhances osteoclast differentiation and bone resorption, whereas OPG functions as a decoy receptor for RANKL, thereby preventing bone loss. The RANKL-to-OPG ratio in GCF is an important marker of bone turnover in the periodontal region. GCF can be collected non-invasively and can be used to monitor the levels of inflammation and bone destruction in periodontal disease.

The integration of these biomarker profiles enables the development of predictive models to assess the risk of systemic disease in patients with periodontal disease.

While numerous biomarkers have been identified in periodontal diagnostics, their clinical utility largely depends on their sensitivity and specificity in detecting and monitoring diseases. Among salivary biomarkers, IL-1β and TNF-α has demonstrated high sensitivity for detecting active periodontal inflammation, making them useful for early detection [141]. However, the specificity of these markers decreases in systemic inflammatory conditions, necessitating the use of additional biomarkers for differential diagnosis. In GCF, the RANKL-to-OPG ratio has shown reliable specificity in indicating bone resorption and periodontal disease progression [142]. For systemic risk assessment, elevated CRP levels, when combined with IL-6, serve as significant predictors of cardiovascular disease [61].

Despite the promising role of biomarkers in periodontal and systemic disease diagnostics, significant challenges hinder their clinical validation and application. One major issue is the variability in biomarker levels across individuals due to genetic differences, lifestyle factors, and the presence of comorbid conditions, which can confound diagnostic accuracy [136]. For instance, elevated CRP levels may indicate systemic inflammation from periodontal disease, but they can also be influenced by unrelated conditions such as obesity or acute infections [132]. Additionally, the lack of standardized cut-off values complicates clinical interpretation, making it difficult to define thresholds for distinguishing healthy and diseased states [141]. Differences in sample collection methods, such as saliva versus blood, further contribute to variability in biomarker levels and their diagnostic utility [143]. Furthermore, most studies validating biomarkers are conducted in controlled research environments, limiting their generalizability to diverse clinical populations [8]. Addressing these challenges requires large-scale, multicenter studies to establish robust biomarker thresholds and validate their sensitivity and specificity across populations. Standardization of protocols for biomarker measurement and data interpretation will also be crucial for translating these markers into routine clinical practice.

## Diagnostic methods

**Fig. 9:**
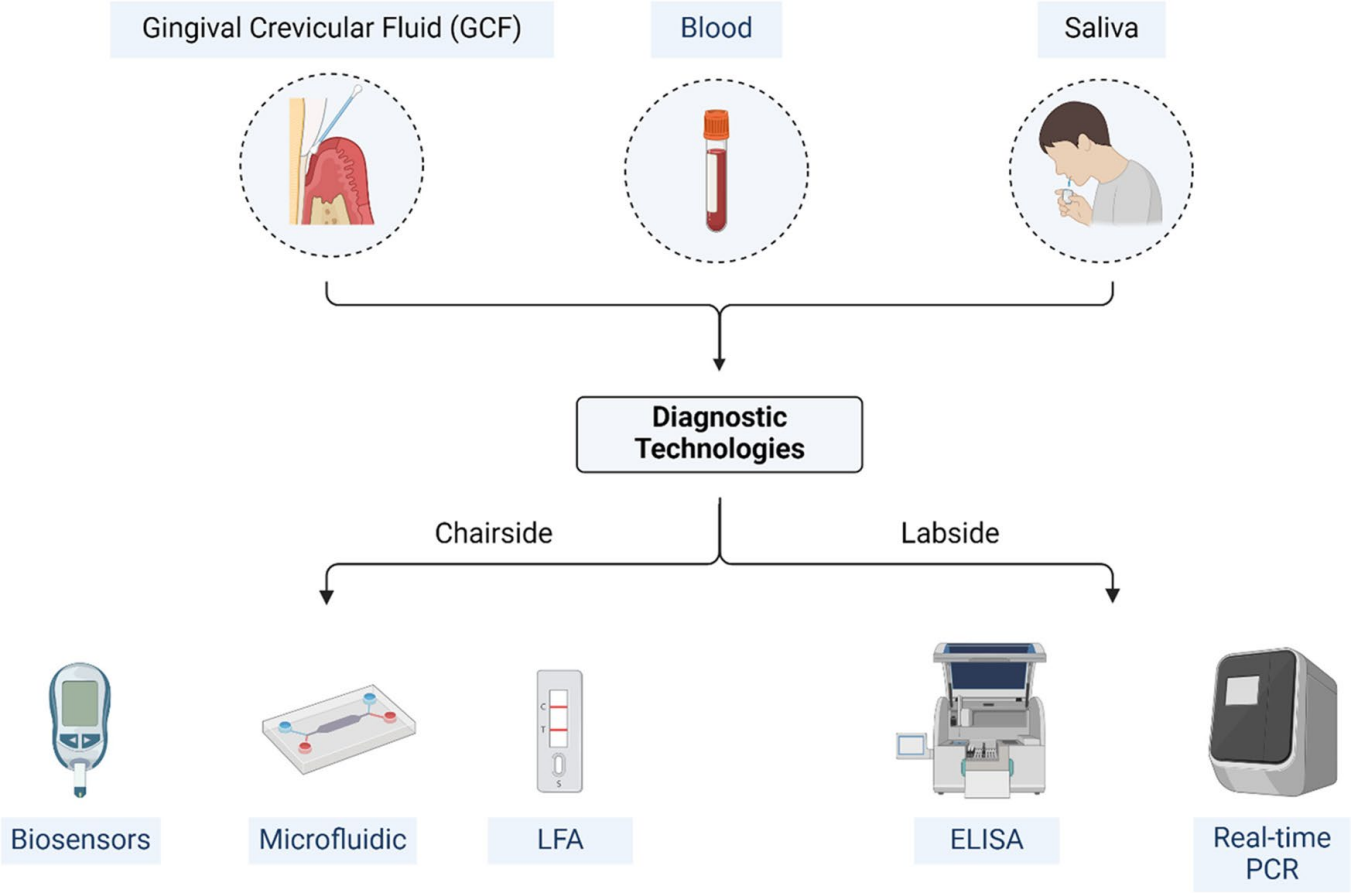
This figure illustrates the use of different sample types (GCF, Blood, and Saliva) in conjunction with modern technologies such as biosensors, microfluidics, and real-time PCR to improve the diagnosis of periodontal diseases. These technologies enable rapid, sensitive, and specific detection of periodontal pathogens and biomarkers, leading to more accurate and timely treatment

Diagnostic technologies can be classified into two primary categories: Labside and Chairside. Labside diagnostics are performed in laboratory settings and are very precise methods, including enzyme-linked immunosorbent assays (ELISA) and polymerase chain reactions (PCR). These methods are generally regarded as the most reliable for biomarker measurement and pathogen detection and are very useful in the diagnosis of periodontal diseases. However, their dependence on specific equipment, long processing times, and high costs mean that they may not be very useful for day-to-day practice in a clinical setting. However, chairside diagnostics can be used in healthcare facilities or even in the comfort of one’s home to offer quick and convenient results. With advancements in lateral flow assays (LFAs), microfluidics, lab-on-a-chip devices, and biosensors, the field of point-of-care testing has evolved considerably. These technologies enhance the ability to measure biomarkers in real-time and enhance the assessment and control of periodontal and systemic diseases. In addition, the implementation of AI in diagnostic devices has improved the effectiveness and reliability of these tools, thereby facilitating the analysis of multiple biomarkers simultaneously with the help of smartphone-based platforms. Thus, both Labside and chairside approaches are mutually beneficial and contribute to the advancement of the diagnostic system for the management of oral and systemic diseases as shown in Fig. 9 and listed in Table 2.

**Table 2:** Diagnostic methods for periodontal disease

Method	Description	Advantages	Disadvantages
Labside			
ELISA	Immunological assay for quantifying proteins (cytokines, enzymes, antibodies)	High specificity, reproducibility, quantitative results	Time-consuming, requires specialized equipment and personnel, high cost
PCR	Amplifies DNA or RNA for pathogen identification	High sensitivity, rapid turnaround time	High cost of reagents and equipment, stringent protocols required
Chairside			
LFAs	Immunochromatographic assay for rapid biomarker detection	Portable, cost-effective, minimal infrastructure required	Lower sensitivity compared to lab-based methods
Microfluidics and lab-on-a-chip	Manipulates fluids within microscale channels for multiplex testing	Small sample volumes, efficient, potential for integration with portable systems	High initial development costs, complex manufacturing
Biosensors	Integrate biological recognition elements with transducers for real-time analyte detection	High sensitivity and specificity, rapid, quantitative results, potential for continuous monitoring	Affected by environmental factors, higher cost

### Labside methods

#### Enzyme-linked immunosorbent assay (ELISA)

ELISA is a widely used immunological method valued for its sensitivity and specificity in detecting proteins such as cytokines, enzymes, and antibodies. In periodontal research, it is particularly effective for analyzing inflammatory biomarkers like IL-1β, TNF-α, and MMPs in saliva, blood, and GCF. These biomarkers provide critical insights into both local and systemic inflammation associated with periodontal diseases and related conditions such as cardiovascular diseases and diabetes [143, 144].

The technique operates on the principle of antibody-antigen specificity, where an enzyme-labeled detection antibody binds to the target biomarker, producing a measurable signal (colorimetric, fluorescent, or chemiluminescent) upon reaction with a substrate. This signal, quantified using a spectrophotometer or fluorescence/chemiluminescence reader, directly correlates with the concentration of the biomarker in the sample. By comparing the results to a standard curve, researchers can accurately quantify inflammation markers, aiding in the assessment of disease severity and treatment efficacy.

While ELISA offers high specificity, reproducibility, and the ability to quantify biomarkers, it has limitations. It is time-consuming, requires specialized equipment and trained personnel, and is therefore best suited for research or centralized laboratory settings rather than rapid diagnostics or point-of-care applications. Despite these challenges, ELISA remains a cornerstone in periodontal research for studying disease pathogenesis and evaluating the impact of treatment interventions [19, 145].

#### Polymerase chain reaction (PCR)

PCR is a molecular technique used for the amplification and measurement of a particular DNA or RNA in the body, and thus is very helpful in isolating periodontal pathogens, including *P. gingivalis*, *T. denticola*, and *F. nucleatum*. PCR is now widely applied in periodontal diagnosis for the detection of microbial DNA in saliva, blood, and GCF samples. This approach provides a view of the structure of biofilms in the mouth and the imbalance in microorganisms, which is the basis of periodontal diseases. Its potential to differentiate between particular bacterial species with a high level of precision is particularly useful in the analysis of disease causation and assessment of treatment efficiency [35, 146].

PCR involves repeated cycles of denaturation, annealing, and extension, during which a specific segment of DNA (or RNA, following a reverse-transcription step) is exponentially amplified. In practical terms, a biological sample (e.g., saliva, blood, or GCF) is first collected and subjected to DNA/RNA extraction to isolate genetic material. The extracted sample is then combined with primers (short DNA fragments complementary to the target region), nucleotides, and heat-stable DNA polymerase. During denaturation, high temperatures separate double-stranded DNA; upon cooling, primers anneal to complementary sequences, and during extension, the polymerase synthesizes new DNA strands. These cycles are repeated multiple times, exponentially increasing the amount of the target DNA. Ultimately, the amplified DNA can be detected via gel electrophoresis or real-time monitoring, providing a high-sensitivity method for identifying periodontal pathogens, such as *P. gingivalis*, *T. denticola*, and *F. nucleatum*. This approach offers rapid detection and accurate differentiation of bacterial species, supporting precise disease assessment and the design of effective treatment plans [147].

The primary advantage of PCR is its high sensitivity, which can therefore help to identify low concentrations of pathogens or their DNA products, and the results can be obtained quickly. Nevertheless, its use is constrained by the expense of reagents and equipment, as well as the protocol that must be followed to avoid contamination, which can affect the results. Such constraints are observed in specialized laboratories with qualified personnel. Nonetheless, PCR is still one of the most important techniques used in periodontal research and diagnosis because it allows for accurate differentiation of pathogenic bacteria and may help in the design of more effective treatment plans [143, 148].

However, the application of ELISA and PCR for periodontal diagnosis has been hindered by challenges related to time, cost, and accessibility. Both techniques are time-intensive and require multiple processing steps, from sample preparation to interpretation, making them unsuitable for rapid diagnostics, where timely clinical decision-making is critical [149]. Their high operational costs, driven by the need for sophisticated instruments and reagents, pose a significant barrier, particularly in low-resource settings and in private dental practices. Accessibility is further limited by reliance on specialized laboratory infrastructure and trained personnel, restricting these methods to centralized facilities and diminishing their utility for point-of-care testing. Additionally, the stability of saliva, blood, and GCF samples as biomarkers and microbial DNA degradation without proper storage is a logistical challenge that complicates diagnostics in remote or underserved areas. Finally, the single parameter focus of traditional ELISA and PCR assays fails to capture the multifactorial nature of periodontal diseases, often requiring multiple tests to obtain a comprehensive and accurate diagnostic profile. [145].

### Chairside methods

#### Lateral flow assays (LFAs)

LFAs are compact devices that are used for point-of-care diagnosis of certain biomarkers through the application of immunochromatographic techniques. These devices rely on antibodies or other binding agents to test proteins, DNA or other targets in body fluids including saliva, blood and GCF. LFAs are especially useful for point-of-care testing because they are easy to use, relatively inexpensive, and do not require much equipment [150]. In periodontal diagnostics, LFAs can measure inflammatory markers, including IL-1β and TNF-α in saliva and GCF, as well as the DNA from *P. gingivalis* and *F. nucleatum*. Quick results, usually obtained within minutes, are useful for the management of periodontal and systemic diseases [151, 152].

Lateral Flow Assays (LFAs) operate through immunochromatographic principles on a porous membrane where a fluid sample (e.g., saliva, blood, GCF) migrates by capillary action [153]. Antibodies or binding agents embedded in the membrane capture target analytes, such as inflammatory proteins (IL-1β and TNF-α) or microbial DNA (e.g., from *P. gingivalis* and *F. nucleatum*). As the sample flows, the labeled detector particles (often gold nanoparticles or colored microspheres) bind to the target analyte, producing visible lines or color changes on the strip. To perform the test, a small volume of the sample is applied to the designated area and the assay left to run for a few minutes. The appearance (or absence) of colored lines in the test region confirms the presence or level of the targeted biomarkers, making LFAs a rapid, user-friendly, and cost-effective choice for point-of-care periodontal diagnostics.

Some companies are currently working on the development of LFA to enhance their use. OraSure Technologies is a market leader in the production of saliva-based diagnostic tools and has developed the OraQuick® platform as an example. Abcam has also produced custom antibody-based LFAs for the detection of biomarkers, and Bio-Rad Laboratories has developed immunochromatographic assays that are ideal for biomarker assessment. Abbott also produced good results with its BinaxNOW® platform, which is a rapid diagnostic tool that can be used in any clinical setting. These advancements demonstrate how LFAs are evolving as effective tools for the diagnosis of oral and systemic diseases.

Although LFAs are very effective in delivering important benefits to users, they are not without flaws. However, their specificity is typically lower than that of laboratory-based assays, such as ELISA or PCR, which makes them suboptimal for clinical use without further development. Nevertheless, their effectiveness in domains such as infectious diseases and pregnancy diagnosis proves that they can be effectively applied to periodontal health. Owing to improvements in materials and detection methods, LFAs can act as a connection between conventional techniques and PCP in real-time, making it easier to implement oral health monitoring in different clinical settings [154, 155].

#### Microfluidics and lab-on-a-chip devices

Microfluidics is a branch of fluid dynamics that deals with the control and management of fluids in channels that are in the micrometer range, which allows for the effective handling of small biochemical samples. When incorporated into lab-on-a-chip technology, microfluidics offers platforms that can integrate several diagnostic functions simultaneously. These devices can be utilized for the measurement of cytokines, such as IL-6, enzymes, such as MMPs, and microbial DNA from pathogens, such as *P. gingivalis*. Through the ability of lab-on-a-chip devices to measure several biomarkers at once, the devices provide a systematic assessment of the inflammatory and microbial processes and are thus suitable for the diagnosis and management of periodontal diseases. The ability to reduce the size and multiplexing of tests eliminates the need for multiple assays, thereby enhancing the efficiency of the diagnostic process [156, 157].

Microfluidics and Lab-on-a-Chip devices manage fluids within microscale channels, enabling simultaneous measurement of multiple diagnostic tests on a single miniaturized platform. In practice, a small sample (e.g., saliva, blood, or GCF) is introduced into the chip, where pumps or capillary forces guide the sample through various reaction chambers and detection zones. This architecture allows for the concurrent analysis of multiple biomarkers, such as cytokines (IL-6), enzymes (MMPs), and microbial DNA (e.g., from *P. gingivalis*), using integrated sensing elements (optical, electrochemical, or others). Through miniaturization and sophisticated fluid control, these devices minimize sample volume requirements, reduce the number of separate essays required, and deliver rapid multiplexed readouts of periodontal health. Despite the challenges related to manufacturing complexity and initial development costs, microfluidic and lab-on-a-chip devices show great promise for point-of-care periodontal diagnostics by providing efficient, comprehensive evaluations of inflammatory and microbial processes in a single, portable platform.

Some companies are advancing microfluidics and lab-on-a-chip technologies, including ThermoFisher Scientific, Illumina, Quanterix, and PerkinElmer, which are working on enhancing microfluidic platforms to improve biomarker detection and microbial analysis. As for lab-on-a-chip devices, Cepheid’s GeneXpert^®^ system is well known for its molecular diagnostic abilities, while Bio-Rad Laboratories is focused on the development of chip-based platforms that are scalable for multiplex testing. Siemens Healthineers has incorporated miniaturized lab systems into their ADVIA Centaur^®^ platforms for biomarker analysis, whereas Fluidigm has developed high throughput integrated fluidic circuits for genetic and biomarker profiling. These companies are good examples of how microfluidic and lab-on-a-chip technologies can transform the diagnosis of periodontal and systemic diseases.

The benefits of microfluidic and lab-on-a-chip devices are not confined to multiplexing. These platforms are associated with low sample inputs, which in turn make them very effective and less likely to cause harm to the patient. This potential can be leveraged with portable systems to support the use of point-of-care diagnostics, thus allowing quick and convenient testing. However, these technologies have some drawbacks such as high initial development costs and complicated manufacturing processes that restrict their usage. More clinical data is needed to confirm the effectiveness and safety of this application in various healthcare contexts. However, the future of microfluidic devices in the management of periodontal diseases is enormous because of their capability to provide a systems approach to understand disease processes thus enabling the development of tailored and convenient diagnostic tools [158, 159].

#### Biosensors

Biosensors are analytical instruments that include a biological recognition element coupled with a physical transducer to provide a specific response to an analyte in real-time. These devices have become useful in periodontal diagnostics owing to their capacity to identify biomarkers and pathogens with high sensitivity and specificity. Using a DNA or antigen approach, biosensors can identify periodontal pathogens, such as Treponema denticola, or monitor inflammatory markers, such as CRP and cytokines, in saliva or blood. This capability enables real-time analysis, thus supporting early diagnosis and intervention of periodontal and systemic diseases associated with oral health [160, 161].

Biosensors function by coupling a biological recognition element (e.g., an antibody, DNA probe, or enzyme) to a physical transducer that converts the binding event between the target analyte and recognition element into a measurable signal (e.g., electrical or optical). In practice, biological fluids such as saliva, blood, or GCF are introduced onto the sensor surface where periodontal pathogens (e.g., Treponema denticola) or inflammatory markers (e.g., CRP and cytokines) selectively bind to the recognition element. This interaction prompts an immediate response detected by the transducer, generating a real-time readout that correlates with analyte concentration. By offering high sensitivity and specificity, as well as the potential for miniaturization and wireless connectivity, biosensors enable rapid point-of-care evaluation of periodontal and systemic health, although their performance may be influenced by environmental conditions and costs compared to traditional assays [162, 163].

The following companies are engaged in the development of biosensors and are pushing boundaries for diagnostic applications. Quanterix boasts that the Simoa^®^ platform offers highly sensitive measurements of inflammatory biomarkers. Thermo Fisher Scientific incorporates biosensor technology into its system for real-time biomarker analysis. Abbott’s i-STAT^®^ systems are examples of advanced portable biosensors used in point-of-care testing, whereas the Draper Laboratory specializes in high-precision biosensors for continuous monitoring. Roche Diagnostics also provides solutions to biosensor-enabled platforms for high-sensitivity molecular diagnostics. With the help of biosensor advancements, these companies are defining new standards for oral and systemic health management.

The advantages of biosensors extend beyond sensitivity and specificity. They also provide rapid and qualitative results and have the potential to be used with portable wireless systems that can enable continuous monitoring, thus making them suitable for decentralized testing and point-of-care. However, their effectiveness may be influenced by factors such as temperature and humidity, and they are relatively expensive compared to conventional assays. Nevertheless, the challenges that exist include the effects of environmental factors such as temperature and humidity, as well as the costs of biosensors being higher than those of traditional assays. Nonetheless, progress in biosensor technology, especially in the development of smartphone-assisted platforms, is creating opportunities for patient-specific and easily accessible diagnostic tools to improve the capacity to manage oral health and its systemic impact [164, 165].

## The case for multiplex rapid diagnostic devices

The present conceptual advances in the interconnection between periodontal disease and general well-being have underlined the usefulness of sophisticated diagnostic procedures. New technologies that include the capability to detect a number of biomarkers simultaneously in a single device present a shift in the approach that can be applied to enhance the management of periodontal and systemic conditions. Through these devices, it is possible to perform integrated assessments that overcome the challenges of conventional diagnostic tools, which are time consuming, expensive, and limited in accessibility, and provide convenient and compact devices for the simultaneous management of health.

### Benefits of integrating multiple biomarker detections into one device

Multiplex rapid diagnostic devices are designed to simultaneously detect and quantify various biomarkers including inflammatory mediators (e.g., IL-6 and TNF-α), microbial DNA (e.g., *P. gingivalis* and *T. denticola*), and metabolic markers (e.g., CRP). This capability eliminates the need for multiple separate tests, significantly reducing the diagnostic time and costs. For periodontal diseases, where the interplay between local and systemic inflammation is critical, such integrated devices provide a holistic view of the disease mechanisms and progression.

Furthermore, multiplex platforms increase diagnostic utility, as they mimic the complex etiology of periodontal diseases and their association with systemic conditions. For instance, simultaneous assessment of inflammatory cytokines, microbial DNA, and bone resorption markers can be useful for establishing a comprehensive risk assessment and early disease classification. This approach not only improves diagnostic efficiency, but also supports personalized treatment planning, as clinicians can base decisions on a broad spectrum of patient-specific data [156, 158].

A crucial feature of these devices is their ability to provide accurate diagnoses, with AI significantly enhancing the performance of multiplex diagnostic systems [166]. Leveraging machine learning algorithms, AI can simultaneously analyze multiple biomarkers and identify trends, aiding in precise and reliable diagnoses. AI also enables the use of prognostic models to assess disease progression and treatment efficacy, thereby enabling clinicians to offer better and more specific care. This integration enables multiplex diagnostic devices to optimize their role in the prevention of periodontal diseases and systemic conditions.

### Potential for user-friendly, portable tools for systemic health monitoring

Integration of multiplex capabilities into portable devices presents a significant opportunity for the advancement of point-of-care diagnostics. These devices are characterized by their small size, and low complexity; hence, they can be used in a number of areas, such as dental clinics and remote health facilities. They provide the convenience of health monitoring at the patient’s bedside or during a standard clinical visit, thus enabling the patient and clinician to act on the information generated in a timely manner. For example, a single device can be used to track the development of periodontal disease as well as other related conditions, including diabetes and cardiovascular diseases.

AI also enhances the usability and effectiveness of these devices and helps with the proper processing of the data. Smartphones are equipped with AI that can connect with platforms that provide real-time results, analysis, and feedback to patients to help them make quick decisions and monitor their health progress at intervals. Such technologies enhance active participation in the management of diseases and increase adherence to recommended treatment plans. From the clinician’s perspective, the application of AI-based solutions helps to accelerate diagnostic procedures, minimize the need for centralized laboratories, and provide monitoring services, which contribute to the enhancement of healthcare delivery in regions with limited access. [151, 159].

## Treatment modalities and their impact on systemic health

Effective treatment of periodontal disease remains a cornerstone in preventing systemic complications, complementing diagnostic innovations critical for early detection. Scaling and root planing (SRP), the gold-standard non-surgical therapy, effectively reduces periodontal inflammation and improves systemic biomarkers, such as CRP and IL-6, which are linked to increased risks of cardiovascular disease and diabetes [19, 20]. For mild cases of gingivitis, non-surgical interventions like professional cleaning and enhanced oral hygiene are sufficient to reverse disease progression [7]. However, in moderate to severe periodontitis, SRP serves as the first-line treatment, targeting the removal of plaque and calculus from subgingival areas [167].

For cases with persistent deep pockets, adjunctive therapies, including localized antimicrobial agents such as chlorhexidine chips and minocycline microspheres, can further target specific periodontal pathogens [168]. Evidence also supports the use of systemic antibiotics, such as amoxicillin and metronidazole, in conjunction with SRP to enhance clinical outcomes in patients with aggressive or refractory periodontitis [167, 168]. In advanced cases with significant tissue or bone loss, surgical interventions, including flap surgery, guided tissue regeneration (GTR), and bone grafting, are indicated to restore periodontal structure and function [169].

Some advancements in treatment approaches, such as laser-assisted periodontal therapy (LANAP), attempt to offer minimally invasive alternatives with promising outcomes in reducing inflammation and promoting tissue regeneration [167, 170]. Additionally, host-modulation therapies, including sub-antimicrobial dose doxycycline (SDD), have demonstrated effectiveness in controlling inflammation and mitigating further tissue destruction [171]. By customizing treatment strategies according to the stage and severity of periodontal disease, clinicians can significantly enhance oral health outcomes and reduce systemic inflammatory burden. This reduction in inflammation can lower the risks associated with cardiovascular and metabolic complications. Implementing a comprehensive approach that integrates early diagnostics with evidence-based treatment protocols ensures improved patient outcomes. Such an approach bridges the gap between periodontal and systemic health, emphasizing the interconnectedness of oral and overall health. By adopting these tailored strategies, healthcare providers can offer more effective and holistic care, ultimately promoting better health and well-being for patients.

## Future directions

As the relationship between periodontal and systemic health has become increasingly evident, there is a paramount need for innovative diagnostic technologies and personalized healthcare solutions. Future advancements in the field will hinge on continued research into device development, biomarker validation, and integration of personalized medicine approaches. These efforts should aim to enhance diagnostic accuracy, streamline disease management, and foster a deeper understanding of the complex interplay between oral and systemic health.

As there is great need for more research in the areas of technology and biomarkers, device development research should focus on enhancing the sensitivity, specificity, and scalability of the devices being developed. Some of the technologies that have been used include microfluidics, biosensors, and lab-on-a-chip devices, which need to be further improved for clinical practice. Some of the major barriers include the need to minimize the costs of manufacturing, while also increasing device reliability and multiple biomarker handling capacity. Additionally, the development of portable and user-friendly devices suitable for point-of-care applications is critical for expanding accessibility, particularly in resource-limited settings [158].

AI is poised to play a revolutionary role in the advancement of diagnostic technologies. AI-driven systems can be used to analyze complex biomarker datasets, identify subtle patterns, and correlate multiple parameters to provide comprehensive diagnostic insights. The use of machine learning in diagnostic devices such as biosensors and microfluidic systems enhances the efficiency and accuracy of devices. In addition, AI is beneficial for enhancing the performance of multiplex devices through real-time analysis of big data, enhanced disease diagnosis and staging, and individualized therapy management. It also plays an essential role in the development of models that can predict disease progression depending on patient characteristics.

Biomarker validation is a critical step for the clinical implementation of these technologies. Although many biomarkers such as IL-6, TNF-α, and P. gingivalis DNA have been identified, confirming their usefulness as a diagnostic and prognostic tool, especially in different populations requires more study. Furthermore, the analysis of changes in biomarkers over time and during the course of the disease and treatment will be useful for setting more accurate diagnostic cutoff points and helping with the decision-making process in clinical practice. Collaboration between researchers, clinicians and industry partners is vital in the process of developing and defining biomarker panels as well as ensuring their reliability in different healthcare settings [143, 156].

The integration of personalized medicine into periodontal diagnostics represents a transformative approach to managing both oral and systemic health. By customizing diagnostics and treatments based on an individual's unique biomarker profile, personalized medicine can significantly enhance therapeutic outcomes and mitigate the risk of systemic complications. For instance, comprehensive biomarker analysis can identify patients at higher risk for cardiovascular disease or diabetes, allowing for early intervention and targeted therapy. This approach not only improves patient care by addressing specific health needs but also fosters a deeper understanding of the interplay between periodontal and systemic health, ultimately leading to more effective and holistic healthcare solutions.

Advancements in digital health and data analytics will facilitate the application of personalized medicine. Smartphone-compatible diagnostic devices and cloud-based data platforms can aggregate and analyze patient-specific health metrics and provide actionable insights in real time. Such systems could enable clinicians to monitor disease progression remotely, optimize treatment plans, and engage patients in proactive health management. Personalized medicine also has the potential to address health disparities by delivering tailored care to underserved populations, ensuring that diagnostic and therapeutic advancements benefit all individuals equally [164, 172].

One of the challenges in the future development of diagnostic technologies is how to address regulatory issues and follow approved pathways. New diagnostic devices, including artificial intelligence-based tools and multiplex platforms, must meet a certain set of standards and regulations of regulatory authorities, such as those in the U.S. Food and Drug Administration (FDA) and European Medicines Agency (EMA). These standards indicate that diagnostic devices are safe, effective, and reliable based on clinical validation and performance studies. In addition, the process of seeking approval requires meeting certain conditions in terms of data integrity, patient safety, and interoperability of the device. It is also important that future research should also include post-market surveillance to assess the effectiveness of the devices in real-world situations for the purpose of enhancement. Collaboration among industry players, regulatory authorities, and academic institutions can help in the approval process, thus enabling the integration of new diagnostic technologies into clinical practice at the right time.

The future of periodontal and systemic health assessment is poised for significant advancements through the development of innovative diagnostic devices, validation of novel biomarkers, and application of personalized medicine principles. These advancements will enable healthcare systems to achieve greater diagnostic precision, facilitate early treatment interventions, and enhance patient outcomes. By focusing on these areas, we can revolutionize the management of periodontal diseases, ensuring that treatments are tailored to the individual needs of the patients. This personalized approach not only improves therapeutic efficacy but also reduces the risk of systemic complications associated with periodontal disease. Moreover, these efforts contribute to a broader understanding of the intricate relationship between oral and systemic health. By disseminating this knowledge, we can foster new paradigms in healthcare organizations that emphasize equity and accessibility. This holistic approach ensures that all patients receive comprehensive care that addresses both their oral and systemic health needs, promoting overall well-being and reducing health disparities. Continued research and collaboration among scientists, clinicians, and healthcare policymakers will be essential for driving these advancements, ultimately leading to a more integrated and effective healthcare system.

## Conclusion

The evidence strongly reinforces the intricate relationship between periodontal diseases and overall systemic health. Driven by microbial dysbiosis and chronic inflammation, periodontitis significantly impacts or is associated with numerous systemic conditions, including cardiovascular disease, diabetes mellitus, adverse pregnancy outcomes, neurodegenerative disorders, and certain cancers. Consequently, managing periodontal health is a critical component of comprehensive patient care aimed at mitigating systemic disease risk. While conventional methods for diagnosing periodontal disease exist, advancements are crucial for facilitating early detection and accurate systemic risk assessment, particularly given the limitations of cost and accessibility with traditional approaches. Emerging diagnostic technologies, such as lateral flow assays, microfluidics, lab-on-a-chip devices, and biosensors, hold significant promise. These tools offer the potential for rapid, point-of-care testing, enabling multiplex biomarker analysis and integration with artificial intelligence for enhanced diagnostic precision and personalized management strategies.

Future efforts must focus on validating key biomarkers, developing robust, cost-effective, and user-friendly point-of-care devices, and integrating these innovations into routine clinical practice. Achieving this requires a multidisciplinary approach, fostering collaboration between dental and medical professionals to address the shared inflammatory pathways. Prioritizing accessibility and affordability in diagnostic development is essential to reduce health disparities. Successfully bridging the gap between oral and systemic health through advanced diagnostics and integrated care will ultimately improve patient outcomes and promote global health equity.

## Data Availability

No datasets were generated or analysed during the current study.
